# An Integrative Volatile Terpenoid Profiling and Transcriptomics Analysis for Gene Mining and Functional Characterization of AvBPPS and AvPS Involved in the Monoterpenoid Biosynthesis in *Amomum villosum*

**DOI:** 10.3389/fpls.2018.00846

**Published:** 2018-06-20

**Authors:** Hong Wang, Dongming Ma, Jinfen Yang, Ke Deng, Meng Li, Xiaoyu Ji, Liting Zhong, Haiying Zhao

**Affiliations:** Research Center of Chinese Herbal Resource Science and Engineering, Guangzhou University of Chinese Medicine, Key Laboratory of Chinese Medicinal Resource from Lingnan, Ministry of Education, Joint Laboratory of National Engineering Research Center for the Pharmaceutics of Traditional Chinese Medicines, Guangzhou, China

**Keywords:** *Amomum villosum*, volatile terpenoids, transcriptomics, bornyl diphosphate synthase, pinene synthase, bornyl acetate

## Abstract

*Amomum villosum*, also known as *Fructus Amomi*, has been used to treat digestive diseases such as abdominal pain, vomiting, and dysentery. Volatile terpenoids are the active metabolites in the essential oil of *Fructus Amomi*. Nevertheless, downstream genes responsible for activating metabolites biosynthesis in *A. villosum* still remain unclear. Here, we report the use of an integrative volatile terpenoid profiling and transcriptomics analysis for mining the corresponding genes involved in volatile terpenoid biosynthesis. Ten terpene synthase (TPS) genes were discovered, and two of them were cloned and functionally characterized. AvTPS1 (AvPS: pinene synthase) catalyzed GPP to form α-pinene and β-pinene; AvTPS3 (AvBPPS: bornyl diphosphate synthase) produced bornyl diphosphate as major product and the other three monoterpenoids as minor products. Metabolite accumulation and gene expression pattern combined with AvPS biochemical characterization suggested that AvPS might play a role in biotic defense. On the other hand, the most active ingredient, bornyl acetate, was highly accumulated in seeds and was consistent with the high expression of AvBPPS, which further indicated that AvBPPS is responsible for the biosynthesis of bornyl acetate, the final metabolite of bornyl diphosphate in *A. villosum.* This study can be used to improve the quality of *A. villosum* through metabolic engineering, and for the sustainable production of bornyl acetate in heterologous hosts.

## Introduction

*Amomum villosum* is a plant of the ginger family which is grown in Southeast Asia and especially in southern China. *A. villosum* is also a Chinese traditional medicinal herb, cultivated for its fruits, which contain highly aromatic seeds (Li et al., 2010). The dried fruit, including seeds, is also known as *Fructus Amomi* (Chinese medicine name: Sharen), which has been used to treat digestive diseases such as abdominal pain, vomiting and dysentery (Chinese Materia Medica, 1999; Pharmacopoeia, 2015). In addition, *Fructus Amomi* has been approved by China Food and Drug Administration and due to its aroma and flavor characteristics, it has been widely used in Chinese cuisine for preparation of food, liquors, and tea.

The desired characteristics of *Fructus Amomi* in medicine and cuisine are mainly attributed to its essential oil, in which three potentially bioactive components were identified: bornyl acetate, borneol, and camphor (Xue et al., 2015). Its most active ingredient, bornyl acetate, has been reported to reduce 5-fluorouracil-induced intestinal mucositis (Zhang et al., 2017). Bornyl acetate is the quality standard of *Fructus Amomi* according to the Chinese Pharmacopeia (Pharmacopoeia, 2015). Moreover, it has been reported that bornyl acetate had antioxidant, anti-inflammatory, antiabortion and anticancer activities (Wang et al., 2011; Kim et al., 2013; Chen et al., 2014; Yang et al., 2014; Li and Wang, 2016).

**FIGURE 1:**
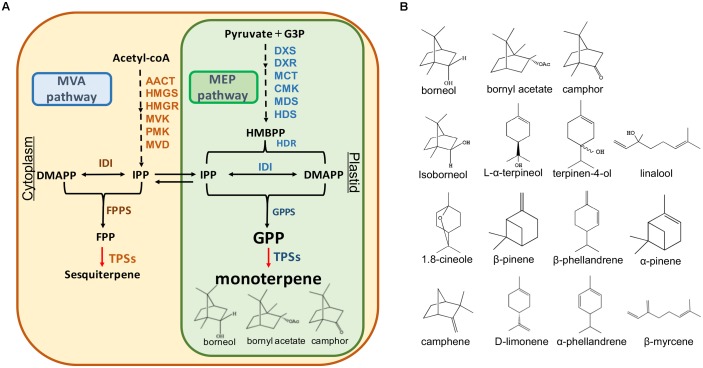
Monoterpene and sesquiterpene biosynthesis pathway **(A)** and the structures of the monoterpenes in *A. villosum* **(B)**. AACT, acetoacetyl-CoA thiolase; HMGS, 3-hydroxy-3 methyl glutaryl coenzyme A synthase; HMGR, 3-hydroxy-3-methyl glutaryl coenzyme A reductase; MVK, mevalonate kinase; PMK, 5-phosphomevalonate kinase; MVD, mevalonate diphosphate decarboxylase; IDI, isopentenyl diphosphate isomerase; FPPS, farnesyl diphosphate synthase; TPS, terpene synthase; DXS, 1-deoxy-D-xylulose-5-phosphate synthase; DXR, 1-deoxy-D-xylulose-5-phosphate reductoisomerase; MCT, 2-*C*-methyl-Derythritol-4- (cytidyl-5-diphosphate) transferase; CMK, 4-(cytidine 5′-diphospho)-2-*C*methyl-D-erythritol kinase; MDS, 2-C-methyl-D-erythritol 2,4-cyclodiphosphate synthase; HDS, 1-hydroxy-2-methyl-2-*(E)*-butenyl-4-diphosphate synthase; HDR, 1-hydroxy-2-methyl-2-*(E)*-butenyl-4-diphosphate reductase; GPPS, geranyl diphosphate synthase.

Terpenoids represent the largest and most diverse class of natural products among the myriad compounds produced by plants (Tholl, 2015). Plant terpenoids are used extensively for their aromatic qualities and have a role in traditional herbal remedies. In plants, there are two metabolic pathways that produce terpenoids: the mevalonate (MVA) pathway in the cytoplasm: and the 2-C-methyl-D-erythritol-4- phosphate (MEP) pathway in the plastid (Tholl, 2015). The upstream steps of these two pathways have been well understood. IPP and DMAPP are the intermediates which produce the monoterpenoid precursor geranyl diphosphate (GPP) and sesquiterpenoid precursor farnesyl diphosphate (FPP) in these pathways (**Figure 1A**).

The complexity and diversity of terpenoids are mainly derived from the highly variable cyclized and/or rearranged nature of the observed hydrocarbon skeletal structures catalyzed by terpene synthases (TPS) (Aubourg et al., 2002; Gao et al., 2012). These synthases convert the acyclic prenyl diphosphates into a multitude of cyclic and acyclic forms. Moreover, terpene diversity arises from the ability of these catalysts to form multiple products from a single substrate (Degenhardt et al., 2009). In addition to the main product, nearly half of all characterized monoterpene and sesquiterpene synthases form significant amounts of additional products (Degenhardt et al., 2009). In *A. villosum*, the bioactive components bornyl acetate, borneol, and camphor belong to monoterpenes. To our knowledge, TPSs responsible for their biosynthesis *in A. villosum* remain unknown.

In recent years, integration of metabolomics and transcriptomics has been developed to explore the biosynthesis pathway of the active ingredients, such as artemisinin, tanshinone, and salidroside in medicinal plants (Gao et al., 2014; Ma et al., 2015; Wei et al., 2016; Torrens-Spence et al., 2018). Over the recent years, dried fruit has been used for the metabolic profiling of *A. villosum* (Deng et al., 2005; Kang et al., 2013; Xue et al., 2015; Zhang et al., 2017). Little is known about the metabolic profiling of fresh tissues (e.g., roots, stems, leaves, seeds, and pericarp) of *A. villosum* growing in its native habitat, Yangchun. This city has been well known for producing genuine and high quality *Fructus Amomi* (Lai et al., 2016). It has been reported that the contents of essential oil and bornyl acetate change with fruit development (Chen and Xu, 2007). In the present study, fruit from two stages, 45 DAF (days after flowering) and 75 DAF, were used for comprehensive metabolomics and transcriptomics to elucidate the complexity of monoterpene and sesquiterpene (essential oil), and to further explore the genes involved in their biosynthesis. Fifteen monoterpenes (**Figure 1B**) were observed by metabolomics of seven fresh tissues of *A. villosum.* In addition, our data revealed higher concentrations of bornyl acetate, borneol, and camphor in nearly mature fruit (75 DAF) seeds compared to young fruit (45 DAF) seeds. The bioinformatic analysis of transcriptomics mined 10 TPS candidate genes (*AvTPS1-AvTPS10*) including five monoterpene synthase genes, three sesquiterpene synthase genes, and two diterpene synthase genes.

Two monoterpene synthase genes with full coding sequences were further cloned and characterized. AvTPS1 catalyzed GPP to produce α-pinene and β-pinene, while AvTPS3 produced bornyl diphosphate as major product, and camphene, β-myrcene, and limonene as minor products. Furthermore, AvTPS3 was highly expressed in the seeds collected at both stages, which was consistent with the accumulation of bornyl acetate, and which suggested that AvTPS3 has an important role in bornyl acetate biosynthesis in *A. villosum*. This study provides a good reference for engineering sustainable production of bornyl diphosphate and the resultant bornyl acetate in heterologous hosts.

## Materials and Methods

### Plant Materials

**FIGURE 2:**
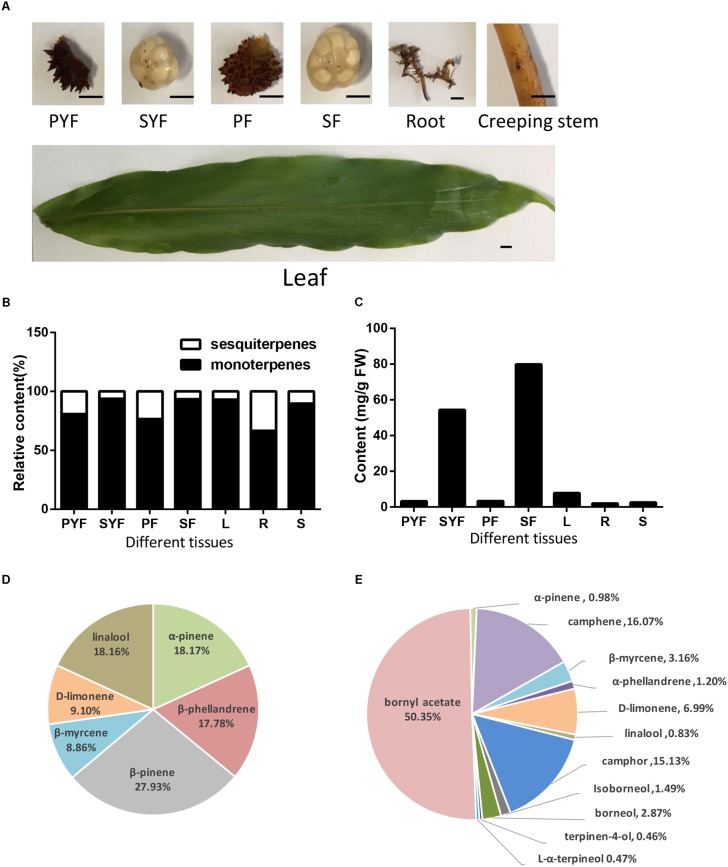
Volatile terpene analysis in different tissues of *A. villosum.* **(A)** The materials diagrams. PYF, pericarp of young fruit (45 DAF); SYF, seeds of young fruit; PF, pericarp of 75-DAF fruit; SF, seeds of 75-DAF fruit; L, leaf; R, root; S, creeping stem. Scale bars = 0.5 cm. **(B)** The ratios of monoterpene to sesquiterpene in different tissues of *A. villosum*. **(C)** The contents of total monoterpenes in different tissues of *A. villosum*. **(D)** Percentage of monoterpenes in the pericarp of 75-DAF fruit. **(E)** Percentage of monoterpenes in the seeds of 75-DAF fruit.

*A. villosum* was planted in Yangchun, Guangdong Province, China. The leaves, stems, roots, creeping stems, and fruits from healthy plants were collected and frozen at -80°C. The fruit was separated into pericarp and seeds. Fruits at different stages were used: YF (young fruit) collected at 45 DAF (days after flowering), with pericarp labeled as PYF (pericarp of young fruit) and seeds labeled as SYF (seeds of young fruit); F (nearly mature fruit) collected at 75 DAF, with pericarp labeled as PF, and seeds labeled as SF (**Figure 2A**).

### Volatile Terpenoid Extraction and Analysis

Approximately, 0.2 g of leaves, roots, creeping stems, and fruits at different stages (separated into pericarp and seeds) were ground frizzed in liquid nitrogen and extracted with 1.5 mL hexane using an ultrasonic cleaner for 30 min, and then incubated at 56°C for 1 h. Samples were then centrifuged at 10,000 rpm for 5 min and the resulting supernatants were pipetted into new 2 mL tubes. One milliliter of hexane extract was pipetted into 1.5 mL vials for GC-MS analysis. The extraction was analyzed using Agilent 7890B Gas Chromatograph with 5977A inert Mass Selective Detector (Agilent, United States). Helium was used as the carrier gas (1 mL/min), and then separated on the HP-5MS column (30 m × 250 μm × 0.25 μm film thickness). GC oven temperature was programmed at an initial temperature of 35°C for 5 min with an increase of 12°C/min to 300°C. Temperature was then kept at 300°C for 5 min. NIST14/Wiley275 Mass Spectral Library was used for metabolite identification. The terpene compounds were identified by the mass spectral library. The predominant monoterpenoids in this research, including α-pinene, β-pinene, camphene, β-myrcene, D-limonene, linalool, camphor, borneol, and bornyl acetate, were further identified using their authentic standards. The contents of bornyl acetate, camphor, borneol, camphene, and limonene were quantified based on their respective standard curves, and the contents of other volatile terpenes were quantified using α-pinene as an external standard. There were three biological replicates and three technical replicates for each tissue.

### RNA Extraction

The total RNA of each sample was isolated using OminiPlant RNA kit (CWbiotech, China), following the manufacturer’s protocol. RNA quality was verified using a 2100 Bioanalyzer (Agilent Technologies, Santa Clara, CA) and checked using RNase free agarose gel electrophoresis. RNA with OD_260_/OD_280_ at 1.8-2.2 was used for further analysis.

### Library Construction, Sequencing, *de Novo* Assembly, and Annotation

Equal amounts of RNA from each sample (leaves, stems, roots, and two stages of pericarp and seeds) were mixed to obtain a single large pool (total amount >20 μg). Next, poly (A) mRNA was isolated and reverse-transcribed to first-strand cDNA. Then, the second-strand cDNA was synthesized and the cDNA fragments were purified and enriched to construct the final cDNA library. The cDNA library was constructed and sequenced on the Illumina sequencing platform (IlluminaHiSeq^TM^ 2000) using the paired-end technology by Gene Denovo Co. (Guangzhou, China). *De novo* assembly and annotation were performed by Gene Denovo Co. as described (He et al., 2018).

### Screening of the Candidate TPS Genes Involved in Volatile Terpenoid Biosynthesis

The first group of candidate unigenes were identified directly by their KEGG annotation and selected by their length and RPKM (reads per kb per million reads) expression value. Besides the unigenes annotated directly by KEGG, the relevant monoterpene synthase (mono-TPS) and sesquiterpene synthase (sesqui-TPS) sequences from other plants (Supplementary Tables S1, S2) were used to re-annotate unigenes involved in volatile terpenoid biosynthesis by local-Blast from transcriptome data. The re-annotated unigenes were selected with the identity >40%, bit score >100, sequence length >800 bp, and RPKM >0.1. The coding sequence of each candidate TPS unigene was translated into an amino acid sequence for further analysis. The multi-sequence alignments and phylogenetic analysis were performed using the neighbor-joining method with the tool MEGA and iTOL^1^. The information of the TPSs from other plants used for phylogenetic analysis was shown in Supplementary Table S3.

### Correlation Analysis of Candidate Mono-TPS Genes and Monoterpenes Based on the Transcriptome Data and the Volatile Terpenoid Profiling

In order to analyze the correlation of candidate genes and volatile terpenoid, the second transcriptome sequencing with three biological replicates for each tissue was performed by Gene Denovo Co using pericarp, seeds and creeping stems collected at different stages which were the same samples used for the volatile terpenoid profiling. The gene expression level was measured by the number of uniquely mapped reads per kilobase of exon region per million mappable reads (RPKM) (He et al., 2018). The spearman correlation coefficients were calculated using gene expression and volatile terpenoid data. Based on the Spearman correlation coefficient (with correlation coefficient > 0.6 and *p* < 0.05), a network map of gene-metabolite was constructed using Cytoscape.

### Full Length Monoterpene Synthase Gene Amplification

Two TPS candidate genes, AvTPS1 (Accession no. MG431984) and AvTPS3 (Accession no. MG763230), were chosen for cloning. The full-length cDNA was amplified using PrimeSTAR Max DNA Polymerase (Takara, China) following the manufacturer’s instructions. The gene-specific primers for AvTPS1 and AvTPS3 were listed in Supplementary Table S4. The PCR conditions used were the following: 98°C, 1 min; 98°C, 10 s, 50–60°C, 15 s, 72°C, 15 s, 30 cycles; 72°C, 5 min. The product was then ligated into pLB cloning vector (Tiangen, China), to produce pLB-AvTPS1 and pLB-AvTPS3 which were consequently transformed into *Escherichia coli* DH5a cells. The sequence alignments of AvTPS1 and AvTPS3 were performed using DNAMAN.

### Prokaryotic Expression and Purification of AvTPS1 and AvTPS3 Protein

The ORF of AvTPS1 and AvTPS3, excluding the N-terminal transit peptide, were ligated into pET32a(+) expression vector using the In-Fusion Cloning Kit (Takara, China). The primers were described in Supplementary Table S4. The positive constructs were then transformed into the competent Rosetta (DE3) cells and the positive colonies were inoculated in the LB media containing 25 μg/mL carbenicillin and 17 μg/mL chloramphenicol at 37 °C till OD_600_ reached 0.4–0.6. The protein was induced by 0.1 mM isopropyl-β-D-1-thiogalactopyranoside (IPTG) and 0.2% arabinose at 16°C for 16 h. The recombinant proteins were purified with NI-NTA resin, following manual recommendations (Qiagen, Hilden, Germany). The purified proteins were dialyzed in PD-10 Desalting Columns (GE Healthcare).

### Enzyme Assay and Product Analysis

For enzyme characterization, the enzyme assays were performed at 100 μL total volume (25 mM HEPES (pH 7.0), 5 mM MgCl_2_, 5 mM DTT) containing 20–60 μg purification protein and excessive GPP, then mixed gently and immediately overlaid with 200 μL hexane. The reaction was incubated at 30°C for 2 h, and then mixed briefly. The mixture was then centrifuged at 12,000 rpm for 5 min to separate the phases. For dephosphorylation, after reacting for 1 h, samples were treated with 1.5 μL alkaline phosphatase (Thermo Fisher, United States) for another hour at 37°C with 200 μL hexane overlaid. The hexane phase was extracted and used for GC-MS analysis. The GC-MS analysis was performed as described above with the oven temperature ramp as 8°C/min instead of 12°C/min to 300°C. NIST14/Wiley275 Mass Spectral Library was used for metabolite identification. Meanwhile, the standards were also utilized for further identification.

### Subcellular Localization

Vector pAN580, which has enhanced green fluorescent protein (EGFP), was chosen to perform the subcellular localization. Based on the cloning vector pLB-AvTPS1 and pLB-AvTPS3, the complete ORFs of AvTPS1 and AvTPS3 without termination codon were inserted into pAN580 vectors to get pAN580-AvTPS1 and pAN580-AvTPS3 vectors, respectively. The primers for the construction of pAN580-AvTPS1 and pAN580-AvTPS3 were described in Supplementary Table S4. For subcellular localization analysis in tobacco protoplast cells, *Nicotiana tabacum* was grown in a greenhouse for 4–6 weeks. The tobacco leaves were then collected and cut into approximately 0.5 mm strips using small sharp razors. Protoplast isolation and vector transfection were performed as previously reported (Nanjareddy et al., 2016). ZEISS LSM 800 with Airyscan (ZEISS, Germany) was used for subcellular localization; EGFP fluorescent signals were visualized with an excitation wave length of 488 nm and an emission wavelength of 509 nm. The red autofluorescence resulting from chlorophylls was captured by an emission wavelength >650 nm. The empty vector pAN580, which has an EGFP-Fusion tag, and the empty protoplast were used as negative controls.

### Quantitative Real-Time PCR

For quantifying two gene transcriptional expressions, the quantitative real-time PCR (qRT-PCR) was performed. RNA extraction and reverse transcription were performed as described above. The primers (qRT-TPS1F/R and qRT-TPS3F/R) for qRT-PCR were designed by Primer Premier 5.0 and were described in Supplementary Table S4. QRT-PCR of AvTPS1 and AvTPS3 were carried out with EvaGreen 2× qPCR Master Mix (ABM, Canada) on CFX96 Real-Time PCR Detection System (Bio-Rad, United States) following the manufacturers’ instructions. Next, the target gene transcript levels were monitored with double reference genes β-actin and TUA as controls for normalization and calculated using the 2^-ΔΔCt^ method. All these experiments were performed with three biological replicates and two technical replicates.

## Results

### The Volatile Terpenoids in Different Tissues of *A. villosum*

The volatile terpenoid profiling of roots, creeping stems, leaves, fruit, seeds and pericarp collected from their native habitat, Yangchun, was examined to improve our understanding of volatile oil biosynthesis and accumulation. The volatile terpenoids among the different tissues and developmental stages of *A. villosum* were detected and analyzed by GC-MS (**Figure 2A**, Supplementary Figure S1 and Supplementary Table S5). In total, 15 monoterpenes and nine sesquiterpenes were found. Monoterpenes (92.25%) dominated the identified components of the essential oil, followed by a lesser portion of sesquiterpenoids (7.75%). Most of the metabolites were detected in the seeds collected at different developmental stages (young and nearly mature stage); 12 monoterpenes and 6 sesquiterpenes, accounting for 4/5 and 2/3 of total monoterpene and sesquiterpene, were found in the SF (seeds collected at 75 DAF). Alpha-pinene and caryophyllene were the most abundant metabolites throughout all the tissues. Monoterpenoids were the dominant volatile terpenoid in all the tissues (**Figure 2B**). The seeds at both stages had higher monoterpene content compared to other tissues. In addition, higher contents of total monoterpene and bornyl acetate were observed in SF (seeds collected at 75 DAF) compared to SYF (seeds collected at 45 DAF) (**Figure 2C** and Supplementary Table S5). β-pinene (27.93%), α-pinene (18.17%), and linalool (18.16%) were the first three principal components in pericarp (**Figure 2D**); yet, bornyl acetate found in seeds had the highest relative content (50.35%) compared to the eleven other monoterpenes (**Figure 2E**).

### Transcriptome Sequencing, Assembly, Annotation, and Functional Classification

Transcriptome sequencing was performed to gain insight into terpenoid biosynthesis. High-throughput sequencing and *de novo* assembly yielded 157,474 contigs and 144,020 unigenes (Supplementary Table S6). The transcriptome data has been submitted to NCBI with the SRA accession number SRP148009. After aligning to the major public databases Nr, COG, Swiss-Prot and KEGG, 72,002 (49.99%) unigenes were totally annotated (**Table 1**). For functional annotation and classification against the COG database, 15,041 unigenes were grouped into 25 classifications (Supplementary Figure S2). Among them, 1,376 unigenes were assigned to the cluster “secondary metabolites biosynthesis, transport and catabolism”. Gene ontology (GO) has three main categories: biological processes, molecular function, and cellular component. In our transcriptomic data, 31,978 unigenes were grouped into 44 GO classifications with 14,456 unigenes assigned to the metabolic processes.

**Table 1:** Summary of annotation.

Value	*Amomum villosum*
Total number of annotated unigenes	72,002
Rate of total annotation	49.99%
Number of unigenes annotated in Nr	70,938
Number of unigenes annotated in Swiss-Prot	53,951
Number of unigenes annotated in COG	26,303
Number of unigenes annotated in KEGG	21,254


**FIGURE 3:**
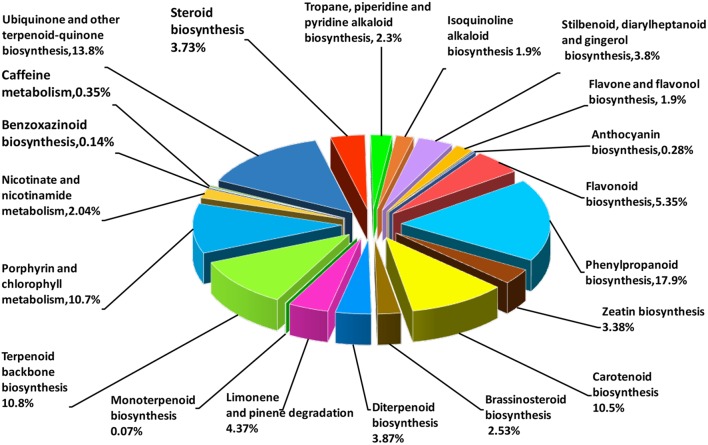
Distribution of unigenes under secondary metabolism categories based on KEGG classifications. Numbers in brackets indicate the relative percentage.

Based on the comparison against the KEGG database, 21,254 unigenes were mapped to 270 KEGG pathways, with 3.73% (the highest percentage) unigenes mapped to “plant hormone signal transduction” (ko04075). In AvM, 21,503 unigenes were mapped to 125 KEGG pathways. To gain insight into secondary metabolic pathways in *A. villosum*, the distribution of unigenes involved in various secondary metabolic pathways was shown in **Figure 3**. Among 18 secondary metabolic pathways, 25.24% unigenes were annotated to be directly involved in terpenoid biosynthesis, including terpenoid backbone, monoterpenoid, diterpenoid, carotenoid (tetraterpene) biosynthesis pathways. Compared with the other transcriptome of *A. villosum* on the MeJA-treatment from our group (He et al., 2018), all the genes involved in terpenoid backbone biosynthesis were annotated in both sets of transcriptome data. For example, *DXS, DXR, MCT, CMK, MDS* were annotated in the MEP pathway, and *AACT, HMGS, HMGR, MVK, PMK* in the MVA pathway; nevertheless more unigenes in total were annotated in this research (Supplementary Table S7).

### Candidate TPS Genes Involved in Volatile Terpenoid Biosynthesis

In order to mine more candidate TPS genes in *A. villosum*, transcriptome data from this research, named AvD, and transcriptome data from MeJA-treated *A. villosum* (He et al., 2018), named AvM, were analyzed. After analysis of unigenes was directly annotated by KEGG, seven mono-TPS unigenes with longer length and higher expression (RPKM) level were selected (Supplementary Table S8). Four of them were annotated to the myrcene or ocimene synthase (MS/OS) gene, while three to the linalool synthase (LIS) gene. Furthermore, the relevant mono-TPS and sesqui-TPS gene sequences from other plants were re-annotated to obtain candidate TPS genes; consequently, unigenes with longer length and higher expression level were selected (Supplementary Table S9). Both annotated and re-annotated unigenes were analyzed and the repetitive unigenes with high identity (>99.6%) between the two sets of transcriptome data were merged. Finally, 10 candidate TPS genes from *AvTPS1* to *AvTPS10* were screened out (**Table 2**). The deduced amino acid sequences of *AvTPS1* to *AvTPS10* were shown in Supplementary Table S10.

**Table 2:** Ten candidate TPS genes.

Name	Unigene		Predicted function
		
	From AvD	From AvM	
*AvTPS1*	Unigene0137026	Unigene0137502	Monoterpene synthase
*AvTPS2*	Unigene0107445	Unigene0134133	
*AvTPS3*	Unigene0115960		
*AvTPS4*		Unigene0136809	
*AvTPS5*	Unigene0047574	Unigene0056791	
*AvTPS6*		Unigene0132051	Sesquiterpene synthase
*AvTPS7*		Unigene0106615	
*AvTPS8*	Unigene0116438	Unigene0106619	
*AvTPS9*	Unigene0093054	Unigene0083374	Diterpene synthase
*AvTPS10*		Unigene0135312	


*AvD, the transcriptome data of this paper; AvM, the transcriptome data of MeJA-treated *A. villosum* (He et al., 2018).*

**FIGURE 4:**
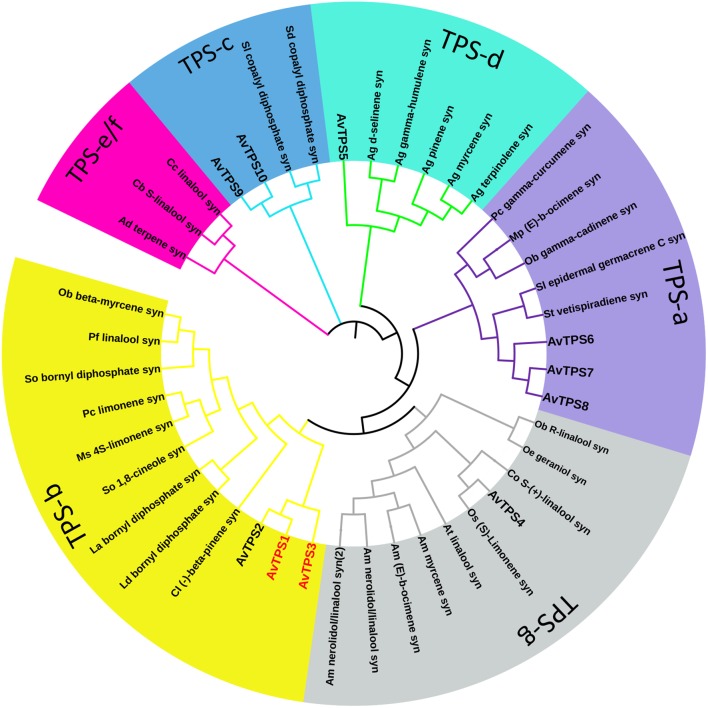
Phylogenetic analysis of AvTPSs from *A. villosum* and the amino acid sequences of other terpene synthases. The terpene synthases from other plants in this analysis were downloaded from NCBI. The detailed information of sequences was show in Supplementary Table S3. The phylogenetic analysis were performed using the neighbor-joining method by the tool MEGA and iTOL (http://itol.embl.de/).

Due to the diversity and specificity of terpenoids as ‘specialized’ metabolites in different plants, TPSs are defined as a mid-size family in the plant kingdom, which include eight subfamilies, from TPS-a to TPS-h; the TPSs responsible for the production of different and special terpenoids are diverse and specific as well (Chen et al., 2011). In this study, the deduced amino acid sequences of 10 AvTPSs were aligned with sequences of already known TPSs from subfamily a-h (Supplementary Table S3) (Chen et al., 2011). In addition, cluster analysis was performed to analyze the relationship between them (**Figure 4**). AvTPS1, AvTPS2 and AvTPS3 were classified into the TPS-b subfamily, which is also specific to angiosperms and mono-TPSs or isoprene synthases, and many of the enzymes of the TPS-b group produce cyclic monoterpenes (Chen et al., 2011). AvTPS4 and AvTPS5 were classified into TPS-g and TPS-d sub-family for mono-TPSs, respectively. AvTPS6, AvTPS7, and AvTPS8 were classified into TPS-a sub-family for sesqui-TPSs. Yet, AvTPS9 and AvTPS10 were classified into TPS-c sub-family for diterpene synthase.

**FIGURE 5:**
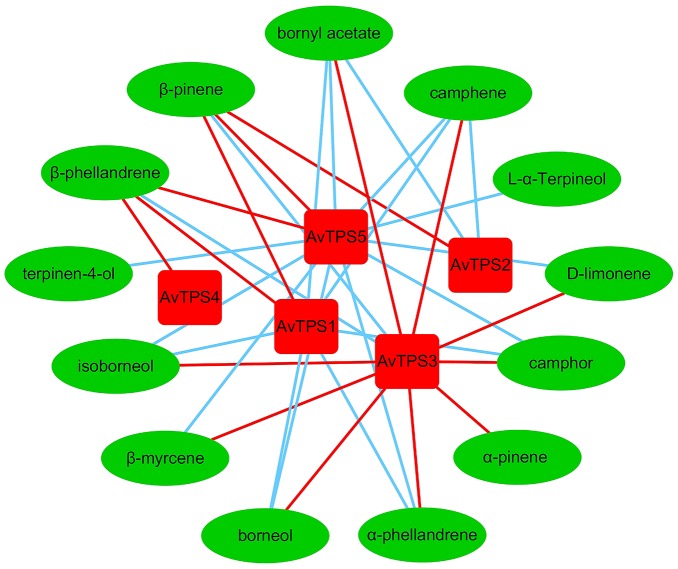
The correlation networks for AvTPS1-AvTPS5 and 13 monoterpenes. The red line represents positive correlation and the blue line represents negative correlation with correlation coefficient >0.6 and *p* < 0.05. The data of the positive correlation was presented in Supplementary Table S11.

Monoterpenoids were the dominant volatile terpenoids in *A. villosum;* bioactive components bornyl acetate, borneol and camphor are monoterpenoids. The fruit, which is the primary medicinal part of *A. villosum*, is abundant with monoterpenes, and both the seeds and creeping stems are rich with bornyl acetate (**Figure 2B** and Supplementary Figure S1). Therefore, two developmental stages of pericarp, seeds, and creeping stems were selected to perform the second transcriptome sequencing with three biological replicates for each tissue to quantify the gene expression. The transcriptome data of five candidate mono-TPS genes and monoterpenoid data were integrated. According to the correlation networks for AvTPS1-AvTPS5 and 13 monoterpenoids (**Figure 5** and Supplementary Table S11), *AvTPS3* was positively correlated with nine monoterpenoids, such as bornyl acetate, borneol, camphor, etc., suggesting that *AvTPS3* was likely responsible for the biosynthesis of these monoterpenoids. In addition, *AvTPS1* and *AvTPS5* were positively correlated with β-pinene and β-phellandrene.

Because AvTPS1 (unigene 0137026) and AvTPS3 (unigene 0115960) have the complete ORFs, they were selected for further cloning and functional characterization primarily. The gene and deduced amino acid sequences of *AvTPS1* and *AvTPS3* have been submitted to GenBank with the accession number MG431984 and MG763230, respectively. Based on the alignment of amino acid sequences, AvTPS1 had the highest identity (87%) with TPS7 (AHJ57305.1) from *Hedychium coronarium*, while AvTPS3 showed highest identity (65%) with a mono-TPS (AHI46572.1) from *Zingiber montanum*; both of them had conserved mono-TPS domains, RRX_8_W, RXR, DDXXD, and (N,D)D(L,I,V)X(S,T)XXXE(NSE/DTE) (Supplementary Figure S3). The motifs DDXXD and NSE/DTE are responsible for metal ion binding and substrate ionization, representing TPS-b class characters. In addition, AvTPS1 and AvTPS3 shared 57% identity.

### AvTPS1 Produced α-Pinene and β-Pinene and Was Renamed as AvPS

**FIGURE 6:**
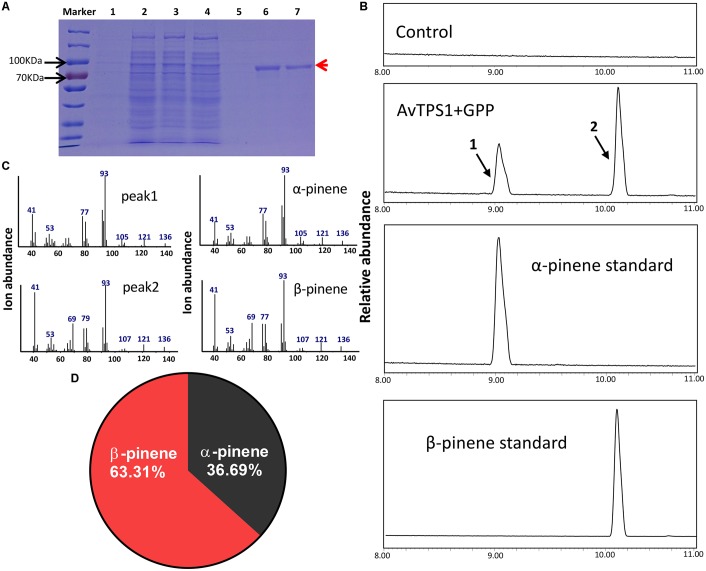
Analysis of reaction products generated by recombinant protein AvTPS1 from GPP. **(A)** Expression and purification of AvTPS1 recombinant protein in *E. coli* Rosetta (DE3) harboring pET32a(+)-AvTPS1. 1, total protein before induction; 2, total protein after induction; 3, soluble protein; 4, no-binding protein; 5–7, purified AvTPS1 recombinant protein from the 1st collected tube to the 3rd collected tube. **(B)** The GC-MS chromatogram of the monoterpene products generated by AvTPS1 protein and the α-pinene and β-pinene standards. The compounds of peaks: 1, α-pinene; 2, β-pinene. **(C)** Mass spectra of peak 1 and peak 2 in the NIST14/Wiley275 library compared with the mass spectra of the α-pinene and β-pinene standards. **(D)** Product percentage of AvTPS1.

The complete ORF (open reading frame) of AvTPS1 was 1803 bp, which encoded 600 amino acids with a predicted 42-amino-acid transit peptide on its N-terminal (**Figure 5**, the red rectangular part). The transit peptide of the monoterpene synthases often reduces the soluble protein expression, so the transit peptide of AvTPS1 was truncated. Then the left coding region was sub-cloned into the pET32a expression vector. The recombinant protein expressed in *E. coli* Rosetta (BL21) expression strain was induced and purified by Ni^2+^ columns. Briefly, SDS-PAGE showed that the recombinant protein of AvTPS1 fused with His and S-tag was about 84.7 kDa as predicted (**Figure 6A**).

In order to verify the AvTPS1 function, the AvTPS1 protein was incubated with GPP or FPP, and the reaction products were detected with GC-MS. With the substrate GPP, AvTPS1 produced two monoterpene compounds, i.e., α-pinene (36.69%) and β-pinene (63.31%) (**Figures 6B–D**). AvTPS1 could not catalyze FPP to form products (data not shown). Therefore, AvTPS1 was renamed as AvPS (pinene synthase). The optimum pH for AvPS was found at pH 6 (Supplementary Figure S4A). The effects of Mn^2+^ and Mg^2+^ on the catalytic activity of the purified proteins were examined as well. AvPS showed much higher activity in the presence of Mg^2+^ than with Mn^2+^ (Supplementary Figure S4C). In addition, kinetic analysis of AvPS with GPP in the presence of Mg^2+^ showed that its Michaelis constant (Km) value was 1.99 μM and its specific constant (K_cat_/K_m_) value was 5.48 × 10^3^ M^-1^S^-1^.

In order to further investigate if AvTPS1 could catalyze GPP to bornyl diphosphate, the dephosphorylation experiment was performed using alkaline phosphatase after reacting with GPP as substrate. The result was consistent with the reaction without dephosphorylation treatment, and only α-pinene and β-pinene were produced (Supplementary Figure S5).

### AvTPS3 Produced Bornyl Diphosphate as Major Product and Was Renamed as AvBPPS

**FIGURE 7:**
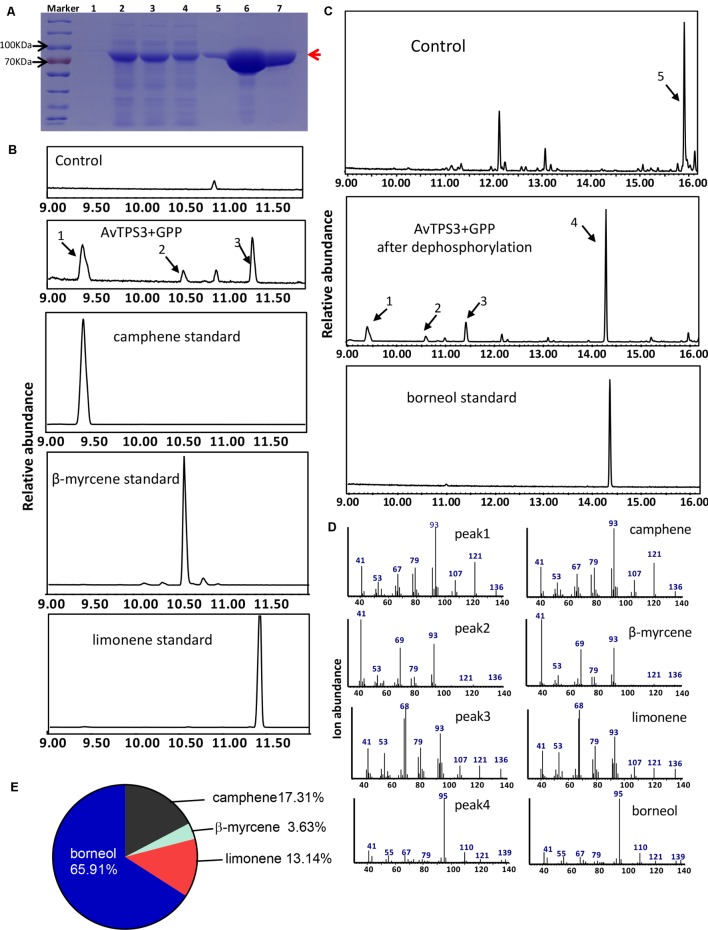
Analysis of reaction products generated by recombinant protein AvTPS3 from GPP. **(A)** Expression and purification of AvTPS3 recombinant protein in *E. coli* Rosetta (DE3) harboring pET32a(+)-AvTPS3 1, total protein before induction; 2, total protein after induction; 3, soluble protein; 4, no-binding protein; 5–7, purified AvTPS3 recombinant protein from the 1st collected tube to the 3rd collected tube. **(B)** The GC-MS chromatogram of the monoterpene products by AvTPS3 protein and the camphene, β-myrcene and limonene standards. **(C)** The GC-MS chromatogram of the monoterpene products generated by AvTPS3 protein after dephosphorylation and the borneol standard. The compounds of peaks: 1, camphene; 2, β-myrcene; 3, limonene; 4, borneol; 5, geraniol. **(D)** Mass spectra of peak 1, peak 2, peak 3 and peak 4 in the NIST14/Wiley275 library compared with the mass spectra of the camphene, β-myrcene, limonene and borneol standards. **(E)** Product percentage of AvTPS3.

The complete ORFs of AvTPS3 was 1791 bp, which encoded 596 amino acids with a predicted 25 amino acid transit peptide on its N-terminal (**Figure 5**, the red rectangular part). The transit peptide was truncated and the left coding region was sub-cloned into the pET32a expression vector. The AvTPS3 recombinant protein fused with His and S-tag was approximately 85.9 kDa (**Figure 7A**).

By using the similar reaction condition and GC-MS method as AvTPS1, AvTPS3 was found to catalyze GPP to produce three monoterpene compounds, camphene, limonene, and β-myrcene (**Figures 7B,D**). After treatment with alkaline phosphatase, bornyl diphosphate transforms to borneol, which can be detected by GC-MS (Despinasse et al., 2017; Hurd et al., 2017). In order to investigate if AvTPS3 could catalyze GPP to bornyl diphosphate, the reacting productions of AvTPS3 with GPP and its negative control were treated with alkaline phosphatase and then analyzed by GC-MS. Four monoterpenes were detected after dephosphorylation; borneol was the major product (65.91%), along with camphene (17.31%), limonene (13.14%), and β-myrcene (3.63%) (**Figures 7C–E**). Therefore, we identified AvTPS3 as bornyl diphosphate synthase (AvBPPS) according to its most abundant product. Like AvPS, AvBPPS was not able to catalyze FPP to form any products. Furthermore, we found that AvBPPS had highest enzymatic activity under pH6 and that it exhibited higher activity with the metal ion Mg^2+^ than with Mn^2+^ (Supplementary Figure S4C). AvBPPS significantly lost its enzymatic activity when the pH value was higher than 7 (Supplementary Figure S4B).

### AvPS (AvTPS1) and AvBPPS (AvTPS3) Localized in Chloroplast

**FIGURE 8:**
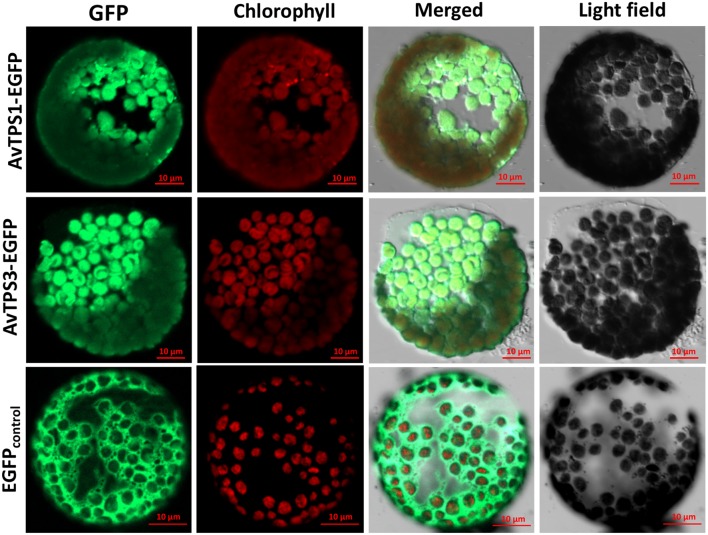
Subcellular localization of AvPS (AvTPS1) and AvBPPS (AvTPS3). Confocal laser scanning microscopy of AvPS and AvBPPS was performed using EGFP-Fusion protein in Tobacco protoplasts. The “GFP” column showed GFP fluorescence detected in the green channel. The “chlorophyll” column showed chlorophyll autofluorescence detected in the red channel, and the “Merge” column showed combined GFP fluorescence and chlorophyll autofluorescence. The name of the fusion vectors were on the left, and the empty vector pAN580 which had EGFP-Fusion tag was used as control.

ChlorpP, WoLF PSORT, Predotar, and TargetP were used to predict AvTPS1 and AvTPS3’s subcellular localization. Briefly, our data showed that they were both localized in plastids with high probability (Supplementary Table S12). To confirm the subcellular localization of AvTPS1 and AvTPS3, pAN580-AvTPS1 and pAN580-AvTPS3 containing the complete ORFs and eGFP-tags were constructed and then transformed into protoplasts of *Nicotiana tabacum* using a PEG-mediated method. The images of protoplast cells were obtained by confocal laser scanning microscopy. The results showed that the green fluorescence of AvTPS1-eGFP and AvTPS3-eGFP were localized in plastid (**Figure 8**), indicating that both AvPS and AvBPPS performed their catalyzing functions in plastid like other mono-TPSs from plants.

### Correlation Between Gene Expression Levels of *AvPS* and *AvBPPS* With Metabolite Accumulation in *A. villosum*

**FIGURE 9:**
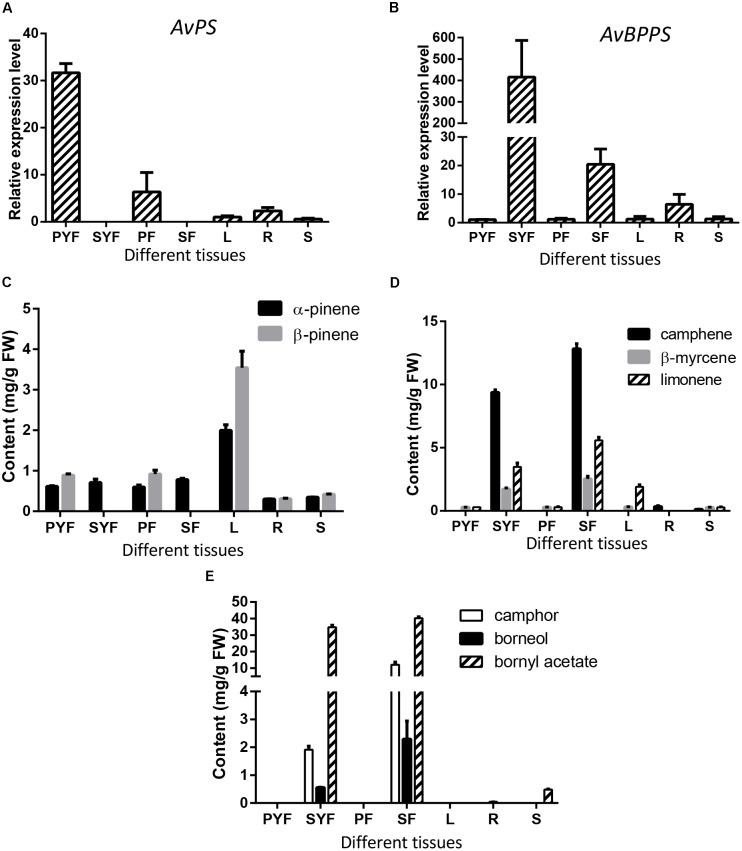
Expression levels of *AvPS* and *AvBPPS* and relative monoterpenes in different tissues of *A. villosum*. **(A,B)** The relative expression level of *AvPS* **(A)** and *AvBPPS* **(B)**. **(C)** The contents of α-pinene and β-pinene in different tissues of *A. villosum*. **(D)** The contents of camphene, β-myrcene and limonene in different tissues of *A. villosum*. **(E)** The contents of camphor, borneol and bornyl acetate in different tissues of *A. villosum*.

To investigate the correlation between the expression levels of *AvPS (AvTPS1)* and *AvBPPS (AvTPS3)* with monoterpene accumulation, qRT-PCR of *AvPS* and *AvBPPS* in seven tissues of *A. villosum* was performed. *AvPS* was highly expressed in the pericarp of both developmental stages (PYF and PF) with the highest expression level in the pericarp of young fruit, but not expressed in the seeds (**Figure 9A**). Interestingly, α-pinene existed in every tissue whereas β-pinene was not found in seeds (**Figure 9B**). Thus, we hypostasized that other pinene synthase(s) might be responsible for the α-pinene biosynthesis in the seeds of *A. villosum*. The ratios of β-pinene to α-pinene in leaves (1.78) and in pericarp of both stages (1.56 in PYF and 1.54 in PF, respectively) were similar to the ratio of β-pinene to α-pinene in the *in vitro* enzyme assay of AvPS (1.73). Nonetheless, among the seven tissues, both α-pinene and β-pinene contents were highest in the leaves, along with a lower expression of AvPS (**Figure 9B**). There might be other TPS that also produce pinene in the leaves of *A. villosum*.

*AvBPPS* was highly expressed in the seeds of both stages (SYF and SF). Interestingly, the expression levels in SYF were approximately 400 times higher than that in leaves (**Figure 9C**). Moreover, the transcript levels in SYF were approximately 20 times higher compared to SF (**Figure 9C**). Borneol is the direct precursor of camphor and bornyl acetate (Adam and Croteau, 1998; Despinasse et al., 2017; Hurd et al., 2017). Therefore, in order to further explore the metabolite accumulation related with AvBPPS expression, the contents of borneol, camphor, and bornyl acetate were analyzed as well as the contents of camphene, limonene, and β-myrcene in different tissues. The results show that the higher expression levels of AvBPPS in SYF and SF were in accord with the higher accumulation levels of camphene, limonene, β-myrcene, borneol, camphor, and bornyl acetate (**Figures 9D,E**). Furthermore, bornyl acetate was much more abundant among the six monoterpene compounds (**Figure 9E**). The higher transcript levels of *AvBPPS* were positive with the higher accumulation levels of camphene, limonene, β-myrcene, borneol, and borneol’s direct products camphor and bornyl acetate in seeds, which was consistent with the *in vitro* enzyme function of AvBPPS.

## Discussion

**FIGURE 10:**
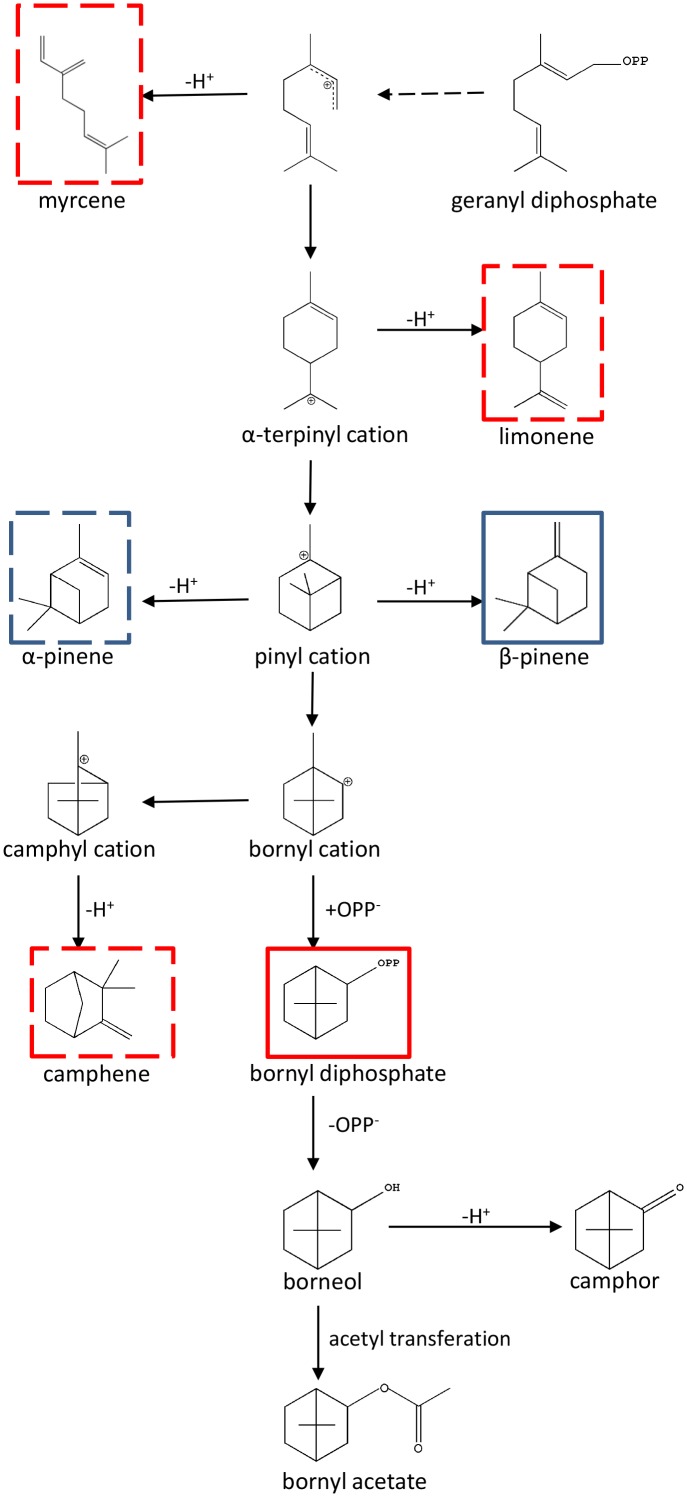
The direct and subsequent products of AvPS and AvBPPS and their downstream pathway. The pathway was adapted from Bohlmann et al. (1997) and Wise et al. (1998). The AvPS products were marked with blue frame and AvBPPS products were marked with red frame. Both main products were marked with full line and the sub-products were marked with dotted line.

Metabolomics is usually referred to as “comprehensive metabolite profiling” and is usually used to identify potential bioactive plant metabolites (Liu et al., 2016; Zhong et al., 2016; Barbosa et al., 2017; Zhu et al., 2017). In *A. villosum*, terpenoid profiling of roots, creeping stems, leaves and two stages of pericarp and seeds were performed to depict the scenario of metabolites among the different tissues and developmental stages. In total, 24 terpenes, including 15 monoterpenes and 9 sesquiterpenes, were found to reveal a tissue specificity trend. This data suggests that the active ingredient production in *A. villosum* is associated with the tissue and its development. Metabolite-related genes were mined through analyzing a combination of metabolomics and transcriptomics data. Ten TPSs genes were screened out; based on the correlation analysis and the integrity of ORF, *AvTPS1* and *AvTPS3* were further selected for cloning and their biochemical functions were investigated. AvTPS1 (AvPS) produced α-pinene and β-pinene, while AvTPS3 (AvBPPS) formed bornyl diphosphate (major product) and camphene, limonene, β-myrcene (minor products). Since bornyl diphosphate is also the precursor of borneol, camphor and bornyl acetate, AvTPS3 (AvBPPS) is responsible for the six main monoterpenes, bornyl acetate, camphor, camphene, borneol, limonene, and β-myrcene in seeds of *A. villosum*. AvPS and AvBPPS were observed to be localized in plastid, which was consistent with their role in monoterpene biosynthesis. The direct and subsequent products of AvPS and AvBPPS and their downstream pathway were shown in **Figure 10**.

According to our terpenoid profiling, monoterpenes and sesquiterpenes were the main volatile components of *A. villous*. Moreover, monoterpenes were more prominent than sesquiterpenes, with monoterpenes accounting for over 76.59 % in pericarp and 93.39 % in seeds of the total terpenoids detected in our samples (**Figure 2B**). Higher monoterpene content in fruit (compared to sesquiterpene) was already reported by previous few studies (Xue et al., 2015; Lai et al., 2016; Zhang et al., 2017). However, in these studies, dried fruit was used for terpene analysis. In contrast, we used fresh samples to depict the real temporal metabolome in *A. villosum*. Our results indicated that the seeds are the main accumulation organ of monoterpene, especially bornyl acetate. There were slight differences between our results and previous reports, where the intact dried fruit was used for measurement (Deng et al., 2005; Kang et al., 2013; Zhang et al., 2017). In our experiment, fruit was divided to pericarp and seeds. Bornyl acetate, borneol, and camphor, the three potentially bioactive components which showed high occurrences in both developmental stages of seeds, were not detected in the pericarp, indicating that these monoterpenes are mainly biosynthesized in the seeds of *A. villosum*.

In our monoterpene profiling, α-pinene was widely distributed throughout almost the whole plant, i.e. roots, creeping stems, leaves, pericarp, and seeds. Interestingly, β-pinene was found in all the tissues, except seeds. Pinene is a naturally occurring constituent of the essential oils in many plant species that has a relevant role in insect repellency and allelopathy (inhibiting root growth of the tested weed species) (Bohlmann et al., 1997; Phillips et al., 1999; McKay et al., 2003; Hassanpouraghdam, 2011; Chowhan et al., 2013; Huang et al., 2013; Pajaro-Castro et al., 2017). AvPS (AvTPS1) catalyzed GPP to form α-pinene and β-pinene, producing 63% β-pinene as a major product. Combined with the fact that AvPS was highly expressed in pericarp, the exterior tissue protecting the fruits against biotic stress, we supposed that AvPS might be the gene involved in biotic defense in *A. villosum*.

*AvPS* (*AvTPS1*) was not responsible for pinene biosynthesis in the seeds of *A. villosum*: no expression of this gene was detected in the seeds, and the expression of *AvPS* was not in accordance with the accumulation pattern of pinene in the leaves; therefore, it was speculated that other pinene synthases were present and were responsible for the pinene biosynthesis of the seeds and leaves, or that other TPSs could produce pinene as one of their multi-products in *A. villosum*. In this study, ten candidate genes were screened. Among them, *AvTPS1* to *AvTPS5* were classified into subfamilies that have mono-TPS function. *AvTPS1* and *AvTPS3* were cloned and characterized first since they had complete ORF in the transcriptome data. Nevertheless, whether *AvTPS2* and *AvTPS5* are involved in pinene biosynthesis needs to be further investigated. The TPS family size in angiosperm plant ranges from approximately 40 to 150 (Chen et al., 2011; Falara et al., 2011; Keilwagen et al., 2017). The TPS members in *A. villosum* should be considerably more than 10 as well. Considering this, besides these 10 candidate TPS genes, other TPS genes in *A. villosum* are likely to be discovered by further research.

Bornyl acetate was also found in other plant species, including liverwort (Adam and Croteau, 1998; Ab Ghani et al., 2016), Korean fir and *Larix decidua* (Pinaceae family) (Jeong et al., 2007; Garcia et al., 2017), *Sabina chinensis* (Cupressaceae family) (Gu et al., 2018), *Lippia sidoides* (Verbenaceae family) (de Morais et al., 2016), *Artemisia montana*, and *Chrysanthemum indicum* (Asteraceae family) (Stoianova-Ivanova et al., 1983; Kunihiro et al., 2017). Bornyl acetate was distributed in the leaves of most of the plants mentioned above, but was especially rich in the seeds of *A. villosum*. AvBPPS (AvTPS3) catalyzed GPP to form camphene, β-myrcene, and limonene, and produced bornyl diphosphate as a major product, which is the direct precursor of borneol. It is thought that borneol is converted to camphor by dehydrogenation (Despinasse et al., 2017; Hurd et al., 2017), and to bornyl acetate by acetyl transferation (Adam and Croteau, 1998). Our correlation analysis indicated that *AvBPPS* (*AvTPS3*) was positively correlated with bornyl acetate, borneol, camphor, camphene, β-myrcene, and limonene (Supplementary Figure S3). Combined with its high expression in seeds, which is consistent with the high content of bornyl acetate, AvBPPS is the key enzyme involved in the bornyl acetate biosynthesis in *A. villosum*. Until now, only three *BPPSs* have been cloned from *Lippia dulcis*, *Lavandula angustifolia*, and *Salvia officinalis* (Wise et al., 1998; Despinasse et al., 2017; Hurd et al., 2017). Alignment of AvBPPS with these three BPPSs showed the relatively lower identity (40% with LdBPPS, 42% with LaBPPS and 41% with SoBPPS). Nevertheless, their mono-TPS domains (RRX_8_W, RXR, DDXXD and NSE/DTE) were highly conserved (Supplementary Figure S6). All four *BPPSs* were clustered into the TPS-b subfamily similarly with the previous report (Despinasse et al., 2017), and *AvBPPS* (*AvTPS3*) rooted the TPS-b clade (**Figure 4**), suggesting that *AvBPPS* might retain more ancestral features than the other three *BPPSs*.

The biosynthesis of bornyl diphosphate, the direct precursor of borneol and bornyl acetate, is the key step for future metabolic engineering and plant breeding. In *A. villosum*, the upstream enzymes HMGR (MVA pathway), DXR and DXS (MEP pathway) in terpenoid biosynthesis have been cloned and characterized (Yang et al., 2012; Wang et al., 2014). Combinational expression of AvBPPS with upstream genes can increase bornyl diphosphate production. An understanding of the gene-terpene network and the key enzymes involved in the biosynthesis of the bioactive components is important for improving the quality of *A. villosum* through metabolic engineering and breeding. The future discovery of the downstream enzymes after AvBPPS, such as dehydrogenase catalyzing bornyl diphosphate to form borneol and acyltransferase catalyzing borneol to form bornyl acetate, could illuminate the complete biosynthesis pathway of bornyl acetate which then could be utilized to synthesize bornyl acetate in heterologous hosts.

## Author Contributions

JY, DM, and HW designed the experiments. HW, KD, ML, XJ, LZ, and HZ performed the experiments and analyzed the data. JY, HW, and DM wrote the manuscript.

## Conflict of Interest Statement

The authors declare that the research was conducted in the absence of any commercial or financial relationships that could be construed as a potential conflict of interest.

## Footnotes

1 http://itol.embl.de/
