# Supplementary material for: An Integrative Volatile Terpenoid Profiling and Transcriptomics Analysis for Gene Mining and Functional Characterization of AvBPPS and AvPS Involved in the Monoterpenoid Biosynthesis in Amomum villosum
Source: Front Plant Sci. 2018 Jun 20;9:846. doi: 10.3389/fpls.2018.00846 (PMC6020762; doi:10.3389/fpls.2018.00846)
Supplement: Supplementary file 1 [file Table_1.PDF]

Supplementary Table 1. Information of Mono-TPS sequences from other plants.

KEGG information			NCBI information		
EC no.	Enzyme	Abbr.	Accession no.	Species	Length/aa
4.2.3.120	(-)-β-pinene synthase	BPS	AAK58723.1	*Artemisia annua*	582
			ADR64206.1	*Artemisia annua*	582
			AFU73843.1	*Pinus banksiana*	628
			AFU73845.1	*Pinus contorta*	627
4.2.3.119	(-)-α-pinene synthase	APS	AFU73855.1	*Pinus contorta*	629
			AFU73856.1	*Pinus banksiana*	629
			AGW25369.1	*Pinus massoniana*	629
4.2.3.108	1,8-cineole synthase	TPS-Cin	AAC26016.1	*Salvia officinalis*	591
			NP_189210.2	*Arabidopsis thaliana*	600
			NP_189212.1		600
			NP_001189976.1		444
5.5.1.8	(+)-bornyl diphosphate synthase	BDS	AAC26017.1	*Salvia officinalis*	598

Supplementary Table 2. Information of Sesqui-TPS sequences from other plants.

KEGG information			NCBI information		
EC no.	Enzyme	Abbr.	Accession no.	*Phyla dulcis*	p.Len/aa
4.2.3.100	bicyclogermacrene synthase	BGS	AFR23369.1	*Artemisia annua*	565
4.2.3.57	β-caryophyllene synthase	BHS	AAL79181.1	*Cucumis sativus*	548
			AAU05952.1	*Mikania micrantha*	567
			ACN67535.1	*Phyla dulcis*	547
			AFR23370.1	*Malus domestica*	555
			AGB14624.1	*Achillea millefolium*	554
			AGZ84810.1	*Ocimum kilimandscharicum*	548
			AKA94109.1	*Artemisia absinthium*	542
			BAN81914.1	*Oryza sativa* Japonica Group	548
—	β-elemene synthase	BES	ABJ16553.1	*Eleutherococcus trifoliatus*	576
4.2.3.133	α-copaene synthase	ACS	ADK94034.1	*Ricinus communis*	556
			AEQ27766.1	*Phyla dulcis*	558
			AFR23368.1	*Mentha x piperita*	564
4.2.3.47	β-farnesene synthase	BFS	AAB95209.1	*Artemisia annua*	550
			AAX39387.1	*Phyla dulcis*	574

Supplementary Table 3. Information of terpene synthases used in phylogenetic analysis.

TPS-subfamily	Terpene synthase	Species	GenBank accession no.
TPS-a	vetispiradiene synthase	*Solanum tuberosum*	BAA82092.1
	epidermal germacrene C synthase	*Solanum lycopersicum*	AAC39431.1
	gamma-curcumene synthase	*Pogostemon cablin*	AAS86319.1
	gamma-cadinene synthase	*Ocimum basilicum*	AAV63787.1
	(E)-B-farnesene synthase	*Mentha x piperita*	AAB95209.1
TPS-b	4S-limonene synthase	*Mentha spicata*	AAC37366.1
	limonene synthase	*Perilla citriodora*	AAG31435.1
	1,8-cineole synthase	*Salvia officinalis*	AAC26016.1
	linalool synthase	*Perilla frutescens*	AAL38029.1
	beta-myrcene synthase	*Ocimum basilicum*	AAV63791.1
	(-)-beta-pinene synthase	*Citrus limon*	AAM53945.1
	bornyl diphosphate synthase	*Lavandula angustifolia*	AJW68082.1
	bornyl diphosphate synthase	*Salvia officinalis*	AAC26017.1
	bornyl diphosphate synthase	*Lippia dulcis*	ATY48638.1
TPS-c	copalyl diphosphate synthase	*Scoparia dulcis*	BAB03594.1
	copalyl diphosphate synthase	*Solanum lycopersicum*	BAA84918.1
TPS-d	terpinolene synthase	*Abies grandis*	AAF61454.1
	gamma-humulene synthase	*Abies grandis*	AAC05728.1
	d-selinene synthase	*Abies grandis*	AAC05727.1
	pinene synthase	*Abies grandis*	AAB71085.1
	myrcene synthase	*Abies grandis*	AAB71084.1
TPS-e/f	S-linalool synthase	*Clarkia breweri*	AAC49395.1
	linalool synthase	*Clarkia concinna*	AAD19839.1
	terpene synthase	*Actinidia deliciosa*	ACO40485.1
TPS-g	R-linalool synthase	*Ocimum basilicum*	AAV63789.1
	geraniol synthase	*Olea europaea*	AFI47926.1
	linalool synthase	*Vitis vinifera*	ADR74215.1
	(S)-Limonene Synthase	*Oryza sativa*	AAR87368.1
	S-(+)-linalool synthase	*Cinnamomum osmophloeum*	AFQ20808.1
	linalool synthase	*Arabidopsis thaliana*	AAO85533.1
	myrcene synthase	*Antirrhinum majus*	AAO41727.1
	(E)-b-ocimene synthase	*Antirrhinum majus*	AAO42614.1
	nerolidol/linalool synthase	*Antirrhinum majus*	ABR24417.1
	nerolidol/linalool synthase 2	*Antirrhinum majus*	ABR24418.1

Supplementary Table 4. Primers used in this study.

name	Primer(5'→3')	Function
AvTPS1-F	TTTTATCCCACATTTACA	The clone of *AvTPS1*
AvTPS1-R	GCTCCATGTAGATAGATTATC	The clone of *AvTPS1*
AvTPS3-F	ATCGATTTGGATGGGTTC	The clone of *AvTPS1*
AvTPS3-R	ATGGCTACCCGTCAAGTAAC	The clone of *AvTPS1*
pET32a-AvTPS1F	AAGGCCATGGCTGATATCATGGCCGAGGAATCTCAATCTCT	The construction of pET32a-*AvTPS1*
pET32a-AvTPS1R	CAAGCTTGTCGACGGAGCTCGAACTGGACAGGTTCAATCAACAACG	The construction of pET32a-*AvTPS1*
pET32a-AvTPS3F	TATCGGATCCGAATTCATGGCCGTGGAGCACC	The construction of pET32a-*AvTPS3*
pET32a-AvTPS3R	GTGCGGCCGCAAGCTTGATTTGGATGGGTTCGAGGAACAG	The construction of pET32a-*AvTPS3*
pAN580- AvTPS1F	CGGAGCTAGCTCTAGAATGTCAGTCTCCCTTTCTTCTGC	The construction of pAN580-*AvTPS1*
pAN580-AvTPS1R	TGCTCACCATGGATCCGAACTGGACAGGTTCAATCAACAACG	The construction of pAN580-*AvTPS1*
pAN580- AvTPS3F	GTCTTAAGTCCGGAGCTAGCTCTAGAATGGCTACCCGTCAAGTAACG	The construction of pAN580-*AvTPS3*
pAN580-AvTPS3R	CCCTTGCTCACCATGGATCCGATTTGGATGGGTTCGAGGAACAG	The construction of pAN580-*AvTPS3*
qRT-AvActinF	GTTCTTAGTGGCGGTTCAA	For reference gene AvActinqRT-PCR
qRT-AvActinR	AGCAGGACCAGATTCTTCAT	For reference gene AvActin qRT-PCR
qRT-AvTUAF	GGAGGATGCGGCAAACAA	For reference gene AvTUA qRT-PCR
qRT-AvTUAR	AGCAAGGAACCCAGCCCAGA	For reference gene AvTUA qRT-PCR
qRT-AvTPS1F	CCAGGCCGAGGGATTATTGA	For AvTPS1 qRT-PCR
qRT-AvTPS1R	TCGTGTGCAGTCTCTGGAAT	For AvTPS1 qRT-PCR
qRT-AvTPS3F	GCGTGGCTTACCACTTCAAA	For AvTPS3 qRT-PCR
qRT-AvTPS3R	TCTGATCTGGTCCTCACAGC	For AvTPS3 qRT-PCR

Supplementary Table 5. Volatile terpenoids in different tissues of *A. villosum.*

Name	Formula	Contents of monterpene and sesquiterpene (ng/mg FW)						
		PYF	SYF	PF	SF	Leaf	Root	Creeping stem
α-pinene	$C_{10}H_{16}$	614.32±18.75	708.38±87.08	596.62±51.44	781.6±34.38	1996.04±137.98	300.31±7.7	346.48±5.16
camphene	$C_{10}H_{16}$	n.d.	9387.47±199.13	n.d.	12837.01±388.73	n.d.	339.35±82.77	128.72±6.83
β-phellandrene	$C_{10}H_{16}$	642.61±16.26	n.d.	583.61±46.39	n.d.	n.d.	304.87±4.47	598.52±22.63
β-pinene	$C_{10}H_{16}$	890.07±30.89	n.d.	916.86±96.61	n.d.	3544.32±407.93	311.53±11.77	419.22±7.41
β-myrcene	$C_{10}H_{16}$	288.24±8.69	1732.47±82.94	290.96±11.57	2525.71±219.46	324.49±5.75	n.d.	277.18±22.44
α-phellandrene	$C_{10}H_{16}$	n.d.	757.66±60.07	n.d.	954.63±33.79	n.d.	n.d.	n.d.
1.8-cineole	$C_{10}H_{18}O$	n.d.	n.d.	n.d.	n.d.	n.d.	309.54±15.6	n.d.
D-limonene	$C_{10}H_{16}$	293.81±5.71	3480.19±314.94	298.78±33.41	5579.83±254.19	1902.69±171.8	n.d.	278.37±36.53
linalool	$C_{10}H_{18}O$	476.46±7.91	494.52±8.99	596.01±55.99	664.9±22.06	n.d.	n.d.	n.d.
camphor	$C_{10}H_{16}O$	n.d.	1912.21±129.49	n.d.	12084.79±1617.11	n.d.	n.d.	n.d.
Isoborneol	$C_{10}H_{18}O$	n.d.	601.78±100.44	n.d.	1193.93±52.32	n.d.	n.d.	n.d.
borneol	$C_{10}H_{18}O$	n.d.	554.53±14.07	n.d.	2290.18±651.31	n.d.	25.52±13.19	n.d.
terpinen-4-ol	$C_{10}H_{18}O$	n.d.	n.d.	n.d.	367.86±5.35	n.d.	351.23±18.2	n.d.
L-α-terpineol	$C_{10}H_{18}O$	n.d.	n.d.	n.d.	376.43±7.55	n.d.	n.d.	n.d.
bornyl acetate	$C_{12}H_{20}O_2$	n.d.	34745.49±1203.88	n.d.	40211.41±869.37	n.d.	n.d.	483.41±20.55
copaene	$C_{15}H_{24}$	316.57±2.93	1092.96±5.95	340.55±9.01	947.74±66.2	n.d.	n.d.	n.d.
α-cubebene	$C_{15}H_{24}$	n.d.	n.d.	n.d.	n.d.	n.d.	317.87±2.56	n.d.
β-farnesene	$C_{15}H_{24}$	n.d.	n.d.	n.d.	662.74±32.1	n.d.	n.d.	n.d.
caryophyllene	$C_{15}H_{24}$	446.25±9.87	n.d.	319.06±14.7	1703.36±118.58	582.53±20.52	348.93±24.29	290.35±0.84
nerolidol	$C_{15}H_{26}O$	n.d.	n.d.	343.7±11.03	n.d.	n.d.	n.d.	n.d.
aromandendrene	$C_{15}H_{24}$	n.d.	779.4±3	n.d.	504.53±12.9	n.d.	n.d.	n.d.
bicyclogermarene	$C_{15}H_{24}$	n.d.	904.37±4.34	n.d.	560.41±18.33	n.d.	n.d.	n.d.
β-bisabolene	$C_{15}H_{24}$	n.d.	801.26±8.02	n.d.	1277.49±37.88	n.d.	n.d.	n.d.
espatulenol	$C_{15}H_{24}O$	n.d.	n.d.	n.d.	n.d.	n.d.	306.42±1.85	n.d.

Volatile terpenes were measured by GC-MS. n.d. means no detection. The results shown are an average of three biological replicates. Metabolite identification was used NIST14/Wiley275 mass spectrum library. The predominant monoterpenoids, including α-pinene, β-pinene, camphene, β-myrcene, D-limonene, linalool, camphor, borneol, and bornyl acetate, were further identified by using their authentic standards.

Supplementary Table 6. Summary of RNA-Seq and Assembly.

Value	*A. villosum*
Q20	97.31%
Total number of clean reads	64,758,162
GC percentage	50.43%
Total number of contigs	157,474
N50 length (bp) of contigs	1,197
Total number of unigenes	144,020
N50 length (bp) of unigenes	1,151
Mean length (bp) of unigenes	714

Supplementary Table 7. KEGG-annotated genes involved in terpenoid backbone biosynthesis pathway.

KEGG number	EC number	Enzyme	Unigene number in AvD	Unigene number in AvM
Unigenes in MVA pathway				
K00626	2.3.1.9	*AACT*	6	5
K01641	2.3.3.10	*HMGS*	5	3
K00021	1.1.1.34	*HMGR*	6	7
K00869	2.7.1.36	*MVK*	6	6
K00938	2.7.4.2	*PMK*	4	4
K01597	4.1.1.33	*MVD*	1	3
Unigenes in MEP pathway				
K01662	2.2.1.7	*DXS*	60	30
K00099	1.1.1.267	*DXR*	7	10
K00991	2.7.7.60	*MCT*	1	2
K00919	2.7.1.148	*CMK*	2	10
K01770	4.6.1.12	*MDS*	2	2
K03526	1.17.7.1	*HDS*	14	26
K03527	1.17.1.2	*HDR*	3	9
K01823	5.3.3.2	*IDI*	2	5
Unigenes for GPP, FPP and GGPP synthesis				
K14066	2.5.1.1	*GPPS*	5	3
K00787	2.5.1.10	*FPPS*	1	1
K13789	2.5.1.29	*GGPPS*	16	11
Total number			141	137

AvD, the transcriptome data of this paper; AvM, the transcriptome data of MeJA-treated *A. villosum* (He et al, 2018).

Supplementary Table 8. Annotated unigenes involved in monoterpenoid metabolism in AvD and AvM.

Description	EC number	KEGG number	Unigene	length/bp	transcriptome
MS/OS	4.2.3.15	K12467	Unigene0047574	711	AvD
			Unigene0056791	938	AvM
			Unigene0056792	955	
			Unigene0056794	968	
LIS	4.2.3.25	K15086	Unigene0060131	450	
			Unigene0060132	976	
			Unigene0136809	1277	

MS/OS,myrcene/ocimene synthase. LIS, (3S)-linalool synthase. AvD, the transcriptome data of this paper; AvM, the transcriptome data of MeJA-treated *A.villosum* (He et al, 2018).

Supplementary Table 9. Re-annotated unigenes involved in monoterpene and sesquiterpene biosynthesis

Pathway	Transcriptome	Unigene	Length/bp	RPKM
Monoterpene biosynthesis	AvD	Unigene0137026	1898	2.9996
		Unigene0115960	2143	18.9919
		Unigene0107445	1573	8.4844
		Unigene0140408	2005	0.4094
		Unigene0093054	2262	2.353
	AvM	Unigene0137502	1018	0.8382
		Unigene0134133	1380	6.6416
		Unigene0135312	2343	0.1222
Sesquiterpene biosynthesis	AvD	Unigene0116438	1883	23.4565
		Unigene0118655	1724	20.2901
		Unigene0082723	935	1.2178
		Unigene0082725	932	2.0173
	AvM	Unigene0132051	2465	5.967
		Unigene0140412	923	50.3158
		Unigene0106615	892	2.8363

AvD, the transcriptome data of this paper; AvM, the transcriptome data of MeJA-treated *A. villosum* (He et al, 2018).

Supplementary Table 10. The deduced amino acid sequences of ten candidate TPS genes.

AvTPS	Amino acid sequence
AvTPS1	MSVSLSSAASATFGSRAGIGGSGSRSAAAIKRRRRLPRIQCHAAEESQSLSSTTS<br>RRSGNYQPSIWTHDRIQSLTLSHAAADEEDHAEEIKLLKYQTSKLMEEKKGRV<br>EEQLQLIDHLQQLGVAYHFKDEIKDTLRGFHASFEDVSLQLRDNLHASALLFR<br>LLRENGFSVSEDIFKKFKDEKAGQFEDRLQSQAEGLLSLYEASYLEKDGEELL<br>HEAREFTTKHLKNLLEEEGSLKPGLIREQVAHALELPLNWRFQRLHTKWFIGA<br>WQRDPAMDPALLGLAKLDFNALQNIYKRELKEASRWWTDLGLPQKLPFFRD<br>RLTENYLWTVGWAFEPDSWAFRELQTKINCFITLIDDVYDVYGTLDELELFTDI<br>MERWDVNSIDKLPEYMKICFLAVFNTVNDAGYEVIRDKGVDIIPYLKRAWAEL<br>CKMYMREARWYHAGYTPTLDEYLDGAWISISGALILSTAYCMGNDLTKDDLD<br>KFSTYPAVMHPSCMLLRLHDDFGTSTDELARGDVQKAVQCCMHERKVPEAVA<br>REHIMQVMEAKWRLLNGNRVATSSFEEYFLNVAINIPRSAQFFYGQGDGYGKS<br>DGETKKQVISLLIEPVQF
AvTPS2	MSLFLAPPSYFPLRSLRRSTAANQPGLPVLVQCSAADKKSPAARRSSPYQPNM<br>WNNDYIQSLTAESPSKGEEEDRTTKRLMLLKERISEVICEKKEVEEQLRLIDHL<br>QQLGVAYHFKDDIKGSLRNFHASLEEISSTFKEDLHATALLFRLLRENGFSITED<br>IFEEFRDEKGHFRDGLNNHAQGMLSLYEASYYEKDGEMVLHEAMEFTTEHLK<br>NLLEEDSADMKLKERASHALELPLNWRMERLHTRWFIEACQREVIVIDNPLLL<br>EFAKLDFNAVQSIYKKELSALSRWWTKLGVVEKLPFVRSRLTENYMWTVGW<br>AFEPEHWSFRDAQTKGNCFVTMIDDVYDVYGNLDELELFTSVVDRWDINAID<br>QLPDYMKILFLALFNTINDDGYKVMKEKGLDVIPYLKRSWADLCKAYLVEAK<br>WYHRGYTPTINGYLENTWIS
AvTPS3	MATRQVTSIYAFPMISVLPRRPMIVTAVEHRGRQTFRRTLQVRSCIATSNVAPLR<br>RSGNYPQNIWTDERVQSLTSTSTEQREEKRERRNVLKEQTRNLILEQQQVAEQ<br>LRLIDHLQQLGVAYHFKDEISDVLSRLHASLDGVSSQLEDDLHATALLFRLLRA<br>NGFSVSQDLFETFRDEKGNFEVRCEDQIRGLLSLYEASYLEKEGEILLKEAMDF<br>ATDKLKGFMEEGSGSLGLREQVAHALQLPLNWRMERVQHRWFIEACNGADD<br>AINPLLLEFAKLDYNLVQDMYKSELRELSSWWSGLGLLEKLPFFRDRLAENYL<br>WAAGFAYEPDSWRCRMIQTKIICLVTMIDDIYDVYGTLDELQLFTDVVDRWD<br>LTAMDKLPEYMKLCFFALFNMVHEEGYRVMKEKGLDIVPDLKRIWGNQCKS<br>YLKEAKWFHHGQIPTLEEYLENGYVSVTTPMVLLHALCAGQDLTGEALKSFS<br>SYYAITRSTGMLFRLYDDMGTSTDEIERGDVAKCIQCYMHEKGVTEEAARKE<br>MTGLMRKYWRESNGYLSWNSPVEEYLKNVAINIPRTAQFFYLYGDGYGMVV<br>DRETKSQIISLFLEPIQI
AvTPS4	MRQARYPVSTDVLHRFHDGRGEFMASLSEDMDGLVNLFEASNLNTGGELILY<br>RVNEFSGHHLESCVAYLEPEFAGGIRQMLEAPSHMTLPRFKARQYLDSDDYSS<br>HGFHLRELGKMDFSLLQSLHQNELKEVTSWWKNSGLGQELPFARDQSLKWF<br>AWIMTCLPNPKFSSYRVVISKIIAFVYLLDDIFDVEGSLEDLHLFAQAIERWDNS<br>SMDSLPNYMRACFEALHSAIDEIAEIVLKEHGWNPIEYLKKSWIQLCNAFLEE<br>AKWLSNDQIPTTDDYMKIATITCGVPAALIHMYFLLGHRPTDDLYDNLPSLISC<br>PARILRLWDDLGSAKDEEQNGRDGSLLACMMKENPNWSLRVAREEVMRMID<br>EAWEELNKESFCSFDSTFSRDFVRACLDSTRMVRVMYSYNEEHNLPLLKEYIN

	LLL
AvTPS5	MEEYLKASLPSISSTTIGLSSLMMMPEAISDEALRFIGPGSRFMHLINLVSRLANDLATFEHEAANGEIASAVSCYQNDHGCTQEEAIAAVEGIIQSSCQELEWELFKSITVVPECYRRGIINIARTSCFVYKRGDSYSLADDDEYATLLKDYLFCPIQ
AvTPS6	MNLVDSIQLLGLDYHFEKEIAAALRSIYEADAANFGLYEVSLRFRLLRQHGYYLSADVFNGFKDDEGRFSSTTLNIGDAKGLLNLYNAAYLGTHGETILDEAIAFTKRQLESLLGELEQPLASEVSLFLETPLCRRIRRLLVRKYIPIYQENPTRNDNILELAKLDFNSLQSLHRKEVKKISTWWKDLALTESLKFARDRVVECYYWIVAVYFEPQYSRARVITTKAISLMSIMDDIYDNYSTLEESRLLTEAIERWEPQAVDHVPEYLKDFYLKLLKTYKDFEDELEPENKYRIPYLLEEIKRLSRSYFQEAKWGVDGYVPLLEEHLPVSLISCGYVAVACAYYVGWGEDATKEAFEWVASFPEILKSCSIICRLMDDITSHEREKERDHHVASTVDSYMKEYGVSSKVALEKLQAMVERAWKDLNKECLHRPTQVARSLIEIIVNLSRAMEDIYKDNDTYTNSNTRMKENVSLVLVQPFPI
AvTPS7	MLYLQDEIKALVISYLQEVKWGIEQHVPSLDEHLQNSLISSGCSTVTCASYVGMGEVVTKEAFDWHSSFPKAIEACCLIGRLLNDIRSHEREQETGHVSSTVESYMKEYGTDAKVACKKLQKIVEKAWKDLNKESLNLTKVPRPIFERIVNFSISMEEIYRYNDMYTKSDTTMKDNISFLLIEPLPM
AvTPS8	MEKQSTTLVASNEERVRKTSKFHPSIWGDYFIHHTSALKKASTYKVVKRREELKEQIKKLFQGTADILQIMNLIDSIQLLGLDYHFEREIDAALHLIFEHDAKNYGLYETSLRFRLLRQHGFYVPADVFNKFKDEEGNFMSTLNEDAKGILSLYNAAYLRIHGEHILDEAILFTKNRFASLLDKLDQPLMTLVYLFLETPLCQRIRRLLTKKYIPIYEEEETRNNTILEFAKLDFNLLQSFHQEELKKISIWWNDLALAKSLTFARDRVVECYYWILNVFFEPQYSHARLISTKVISLLSLMDDIYDNYSTLQESQLLTAAIQRWEPRAVDEVPEYLKDFYLKLLRTFKEFENELESDEKYRVSFLQDEVKALSRSYFLEAKWGIEKYVPTLEEHLNNSLITCGYRVLTCASYVGMGQVATKEAFEWVAGFPKILKASSLICRLVDDIMSHKREQERGYTATTVECFMKQYATDEKVAYKNLMDMVEDAWKDHNEECLNPTQVARPLIERIVNFSRTIGEFYKYDDTYTNSKTTMKDNVCMVLVESVPI
AvTPS9	MAMTPTTAMTSTLLPPSPLLLLPGGARELRSIGTGKAEKSSRRHALTRTSTTAPPPDYGGGLIQNALPLLELPQQEHVLLEEEESLLQGTTIVGLVQELKAMLGSMEDGEISVSAYDTAWVALAKDPERSERPLFPESLRWIANNQLGDGSWGDAAVFSAHDRLINTLACAVVLASWNLHRDKCLRGVEYVRENMWRLGEEAAEHMPIGFEVAFPSLLDMAKELGLEIPYSHPCLQSIIAMRDLKLKRIPKQVLHEVPTTLLHSLEGMVGLDWEKLLRLQCQDGSFLFSPSSTAYALMQTGDGNCLKYLQRIVRRFGGGVPNVYPVDLFERLWAVDRLQRLGIARYFSPEIKDCLDYVHRYWTEDGICWARDSLVFDIDDTSMGFRLLRLHGYPVSPDVLQQFEKDGEFVCFPGQSNQAVTGMYNLNRAAQVAFPGEEILERAKSFSYAFLRDKQAAHQLLDKWIITKDLPGEVEYALNFPWYASLPRIEARLYLEHYGGGSDIWIGKTLYRMPLVNNDVYLELAKLDFNHCQALHQLEWLLLQKWYDEAGLRRHGVSRRTLLEDYFLAASCIFEPERKTERLGWVRTLVFSKAIAAYFGSDSCTETMRQALILNFLNADDCYSNENGTRRAGTRGKGEQLVELLRQLVDGLVVLGAESDRKRMRDYLLEAWNAWLSTSTGDEQDTGLLLVRTIEACGGQFDSTLARSHCDLLSSICARLLQRKLDAKSTDKETIDE

	KMQELAQCVLQSSSSSSSSSEEQGIDLRRTKQTFLLVAKSFYYAAHCSEDVLN
AvTPS10	MAMTPTTAMTSTLLPPSPLLLLPGGARELRSIGTGKAEKSSRRHALTRTSTTAP PPDYGGGLIQNALPLLELPQQEHVLLEEEESLLQGTTIVGLVQELKAMLGSME DGEISVSAYDTAWVALAKDPERSERPLFPESLRWIANNQLGDGSWGDAAVFSA HDRLINTLACAVVLASWNLHRDKCLRGVEYVRENMWRLGEEAAEHMPIGFE VAFPSLLDMAKELGLEIPYSHPCLQSIIAMRDLKLKRIPKQVLHEVPTTLLHSLE GMVGLDWEKLLRLQCQDGSFLFSPSSTAYALMQTGDGNCLKYLQRIVRRFGG GVPNVYPVDLFERLWAVDRLQRLGIARYFSPEIKDCLDYVHRYWTEDGICWA RDSLVFDIDDTSMGFRLLRLHGYPVSPDVLQQFEKDGEFVCFPGQSNQAVTG MYNLNRAAQVAFPGEEILERAKSFSYAFLRDKQAAHQLLDKWIITKDLPGEVE YALNFPWYASLPRIEARLYLEHYGGGSDIWIGKTLYRMPLVNNDVYLELAKLD FNHCQALHQLEWLLLQNFIQLLNLFEFQLCDSTKVVRRGRPSAARCQQKNPV GGLLLSSLLHIRARTQDREARLGSDPGFLQGHRSLLWQ

Supplementary Table 11. The positive correlation of AvTPS1-AvTPS5 and 13 monoterpenes.

Monoterpenoid	Candidate Gene	Correlation coefficient
α-pinene	*AvTPS3*	0.70[a]
camphene	*AvTPS3*	0.63[b]
β-phellandrene	*AvTPS1*	0.78[a]
	AvTPS4	0.69[a]
	AvTPS5	0.90[a]
β-pinene	*AvTPS1*	0.84[a]
	AvTPS2	0.74[a]
	AvTPS5	0.86[a]
β-myrcene	*AvTPS3*	0.74[a]
α-phellandrene	*AvTPS3*	0.75[a]
D-limonene	*AvTPS3*	0.76[a]
camphor	*AvTPS3*	0.75[a]
isoborneol	*AvTPS3*	0.75[a]
borneol	*AvTPS3*	0.75[a]
bornyl acetate	*AvTPS3*	0.63[b]

$^{a}P < 0.01$, $^{b}P < 0.05$.

Supplementary Table 12. Localization prediction of AvTPS1 and AvTPS3 using different software tools.

gene	ChlorpP	WoLF PSORT	Predotar	TargetP
AvTPS1	Chloroplast	Chloroplast	Plastid	Chloroplast
AvTPS3	Chloroplast	Chloroplast	Possibly plastid	Chloroplast
