# Supplementary material for: An Integrative Volatile Terpenoid Profiling and Transcriptomics Analysis for Gene Mining and Functional Characterization of AvBPPS and AvPS Involved in the Monoterpenoid Biosynthesis in Amomum villosum
Source: Front Plant Sci. 2018 Jun 20;9:846. doi: 10.3389/fpls.2018.00846 (PMC6020762; doi:10.3389/fpls.2018.00846)
Supplement: Supplementary file 2 [file Image_1.PDF]

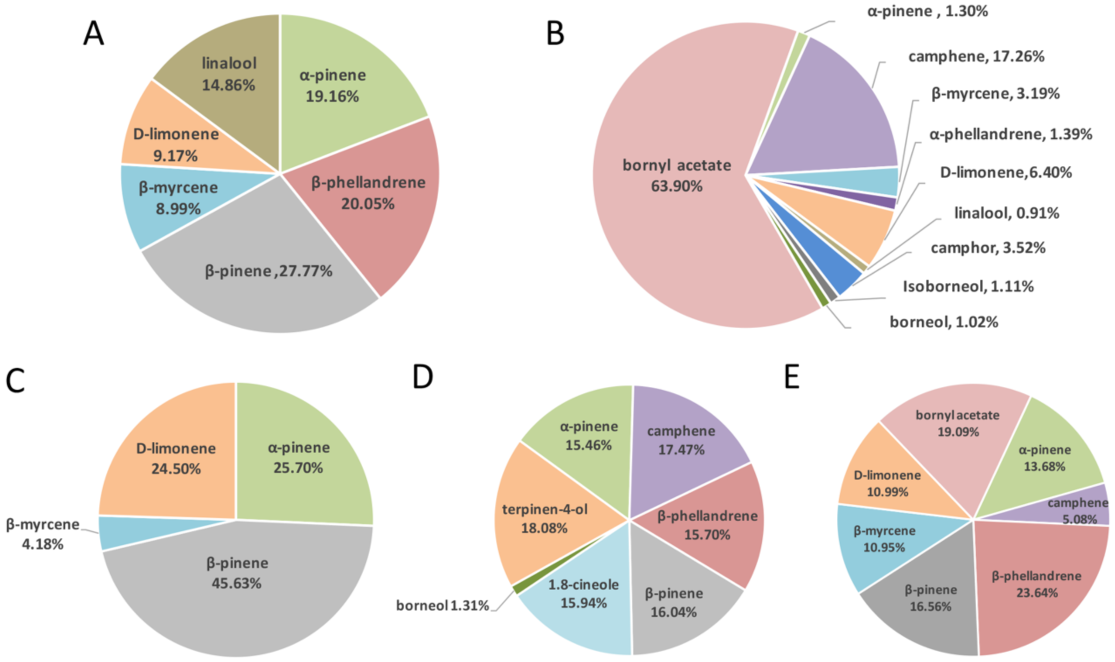

Supplementary Figure 1. Percentage of monoterpenes in different tissues of *A. villosum*.
(A) Pericarp of 45-DAF fruit. (B) Seeds of 45-DAF fruit. (C) Leaf. (D) Root. (E) Creeping stem.

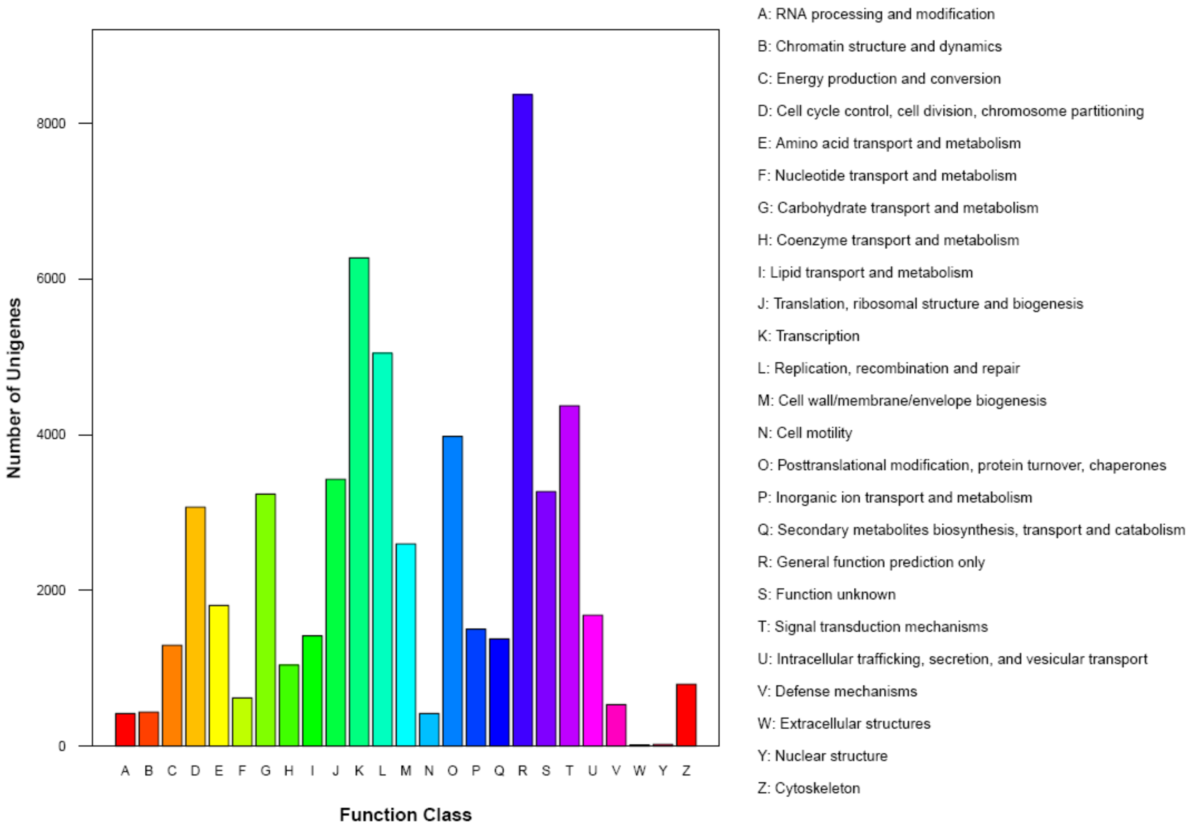

Supplementary Figure 2. COG function classification of *Amomum villosum* unigenes

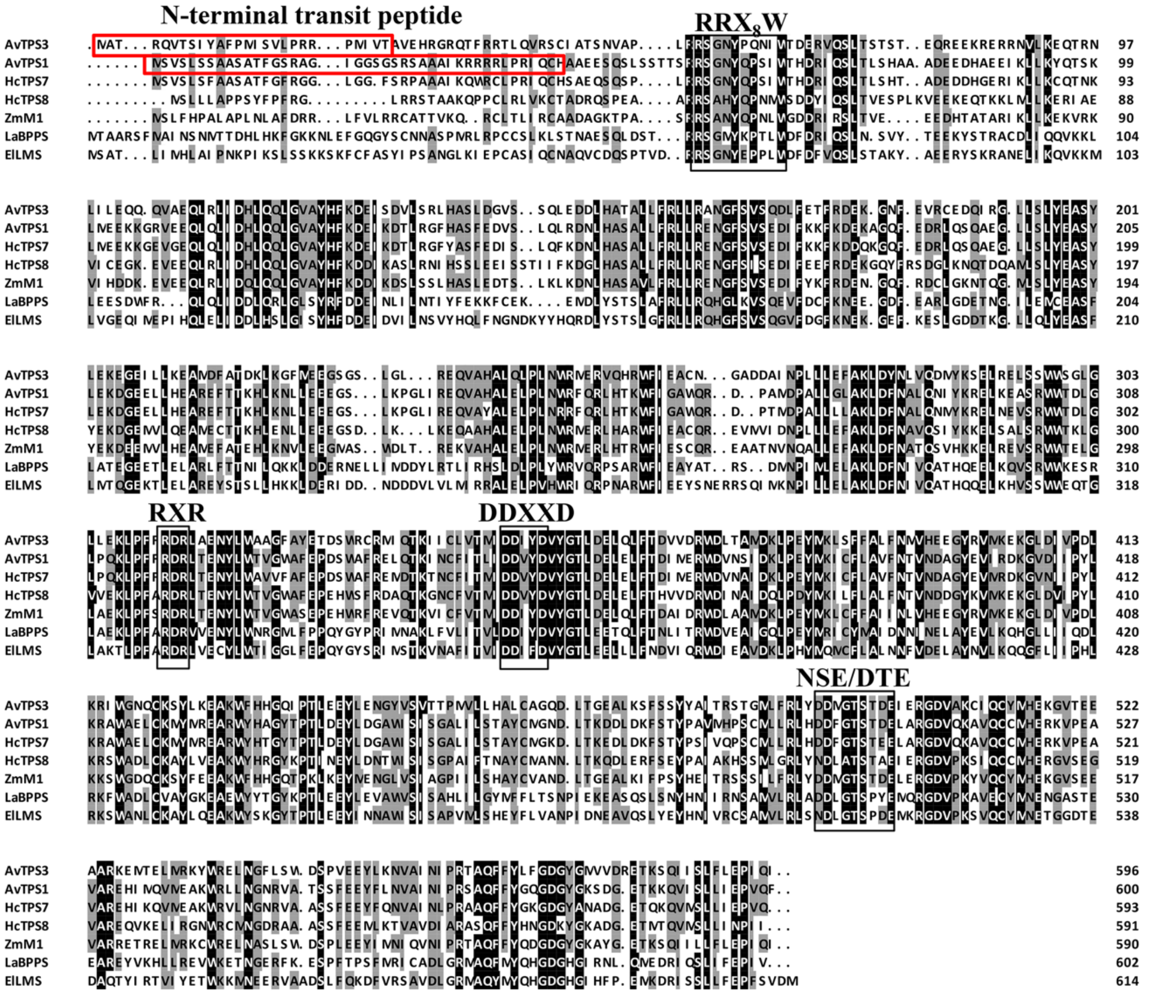

Supplementary Figure 3. Protein alignment of AvTPS1 and AvTPS3 with *Hedychium coronarium* TPS7 (AHJ57305.1), TPS8 (AGY49283.1), *Zingiber montanum* monoterpene synthase (AHI46572.1) and *Lavandula angustifolia* bornyl diphosphate synthase (AJW68082.1) and *Erythranthe lewisii* limonene/myrcene synthase (AHI50308.2) . The predicted transit peptides of AvTPS1 and AvTPS3 were showed with red frame. The conserved domains RRX8W, RXR, DDXXD and NSE/DTE were showed with black frame.

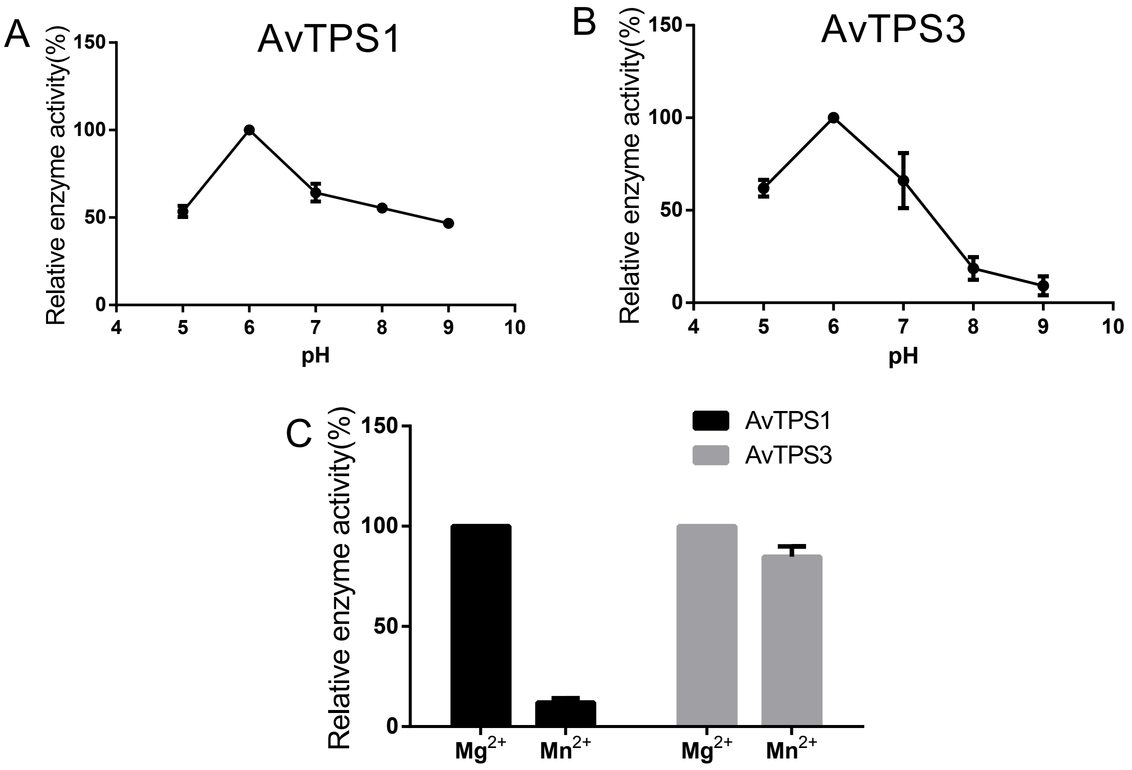

Supplementary Figure 4. The analysis of optimum pH and metal ion in AvTPS1 and AvTPS3. (A) pH analysis of AvTPS1. (B) pH analysis of AvTPS3. (C) Metal ion analysis of AvTPS1 and AvTPS3.

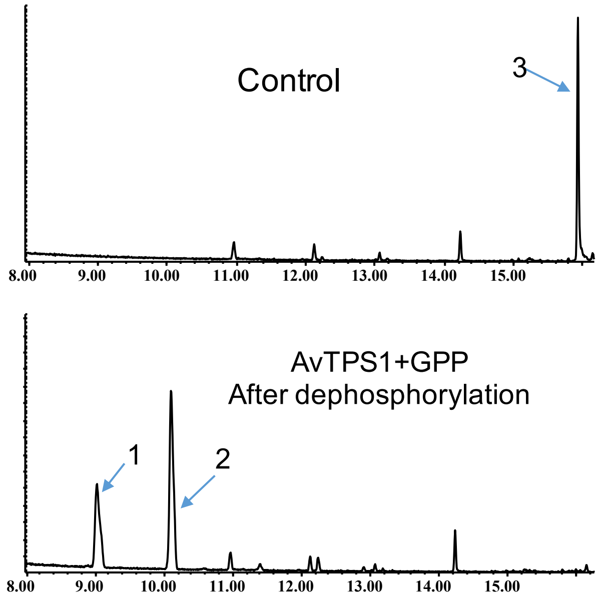

Supplementary Figure 5. The GC-MS chromatogram of the monoterpene products generated by AvTPS1 protein after dephosphorylation. The compounds of peaks: 1, α-pinene. 2, β-pinene. 3. geraniol.

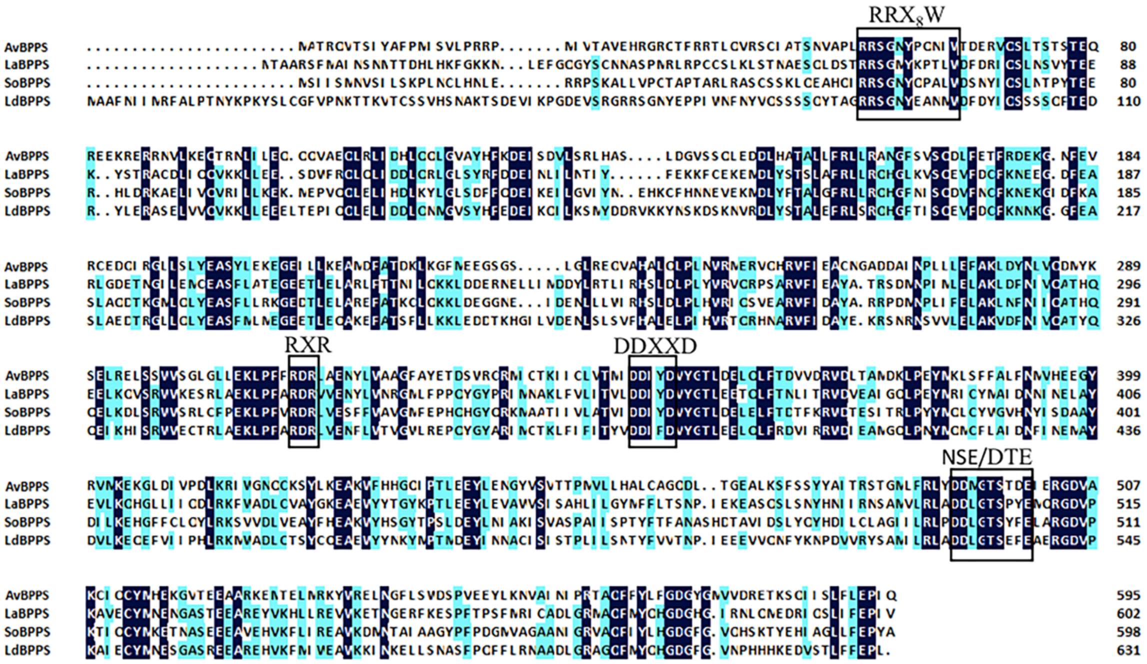

Supplementary Figure 6. Protein alignment of AvBPPS with bornyl diphosphate synthases from *Lavandula angustifolia* (LaBPPS, AJW68082), *Salvia officinalis* (SoBPPS, AF051900) and *Lippia dulcis* (LdBPPS, ATY48638). The conserved domains RRX_8W, RXR, DDXXD and NSE/DTE were showed with frame.
